# Efficacy of Probiotics for Irritable Bowel Syndrome: A Systematic Review and Network Meta-Analysis

**DOI:** 10.3389/fcimb.2022.859967

**Published:** 2022-04-01

**Authors:** Tao Zhang, Cunzheng Zhang, Jindong Zhang, Feng Sun, Liping Duan

**Affiliations:** ^1^ Department of Gastroenterology, Peking University Third Hospital, Beijing, China; ^2^ China Center for Evidence Based Medical and Clinical Research, Peking University, Beijing, China; ^3^ Institute of Public Health, Peking University, Beijing, China

**Keywords:** irritable bowel syndrome, probiotics, network meta-analysis, efficacy, adverse events

## Abstract

**Background:**

Irritable bowel syndrome (IBS) is a common gastrointestinal condition. Studies regarding the treatment of IBS with probiotics have not yielded consistent results, and the best probiotics has not yet been confirmed. Therefore, we performed a network meta-analysis (NMA) to assess the relative rank order of different probiotics for IBS.

**Method:**

We searched for RCTs on the efficacy of probiotics for IBS until August 25, 2021. The primary outcome was the symptom relief rate, as well as global symptoms, abdominal pain, bloating, and straining scores. The NMA was conducted using Stata 15.0. We also used meta-regression to explore whether the treatment length and dose influenced the efficacy.

**Results:**

Forty-three RCTs, with 5,531 IBS patients, were included in this analysis. Firstly, we compared the efficacy of different probiotic species. *B.coagulans* exhibited the highest probability to be the optimal probiotic specie in improving IBS symptom relief rate, as well as global symptom, abdominal pain, bloating, and straining scores. In regard to the secondary outcomes, *L.plantarum* ranked first in ameliorating the QOL of IBS patients, but without any significant differences compared with other probiotic species in standardized mean differences (SMD) estimates. Moreover, patients received *L.acidophilus* had lowest incidence of adverse events. The meta-regression revealed that no significant differences were found between participants using different doses of probiotics in all outcomes, while the treatment length, as a confounder, can significantly influence the efficacy of probiotics in ameliorating abdominal pain (Coef = -2.30; p = 0.035) and straining (Coef = -3.15; p = 0.020) in IBS patients. Thus, we performed the subgroup analysis on treatment length subsequently in these two outcomes, which showed that efficacy of *B.coagulans* using 8 weeks ranked first both in improving the abdominal pain and straining scores. Additionally, *B. coagulans* still had significant efficacy compared to different types of probiotic combinations in present study.

**Conclusions:**

The findings of this NMA suggested that *B.coagulans* had prominent efficacy in treating IBS patients, and incorporating *B.coagulans* into a probiotic combination, or genetically engineering it to amplify its biological function may be a future research target to treat IBS patients. With few direct comparisons available between individual therapies today, this NMA may have utility in forming treatment guideline for IBS with probiotics.

## Introduction

Irritable bowel syndrome (IBS) is a common and chronic gastrointestinal (GI) condition characterized by abdominal pain, bloating, and changes in bowel habits associated with altered stool form, which can affect the quality of life and work productivity of patients (Mearin et al., 2016; Camilleri, 2021). In terms of clinical epidemiology, the prevalence of IBS varies substantially among different countries and different diagnostic criteria, ranging from 1.1% to 45% (Black and Ford, 2020); Furthermore, there is a higher prevalence of IBS in women than in men (12% vs. 8.6%) (Oka et al., 2020). IBS can be diagnosed by reviewing the clinical findings based on the Rome Criteria rather than basing the diagnosis on definite biological markers and organic lesions in patients with IBS (Lacy and Patel, 2017).

There are trillions of microbes residing in the human GI tract, which is over 150 times the number of genes in the human genome (Qin et al., 2010; Raskov et al., 2016). Beneficial commensal bacteria, which play an important role in healthy individuals, can contribute to the upregulation of anti-inflammatory genes and downregulation of pro-inflammatory genes (Plaza-Diaz et al., 2014). In IBS cases, the reduction of microbiome diversity, gut barrier deficiency, gut-brain signaling disorders, and immune disorders are significantly related to the abnormal function of the GI tract (Raskov et al., 2016). Liu et al. (2016) found that when compared with healthy controls, the diarrhea predominant IBS (IBS-D) group had lower biodiversity of microbial communities, which were dominated by *Bacteroides* and *Prevotella* genera. Moreover, a decrease in probiotic species and an increase in pathogenic species were also found to be common in IBS cases (Ringel and Ringel-Kulka, 2015).

Probiotics, available in various dietary components or by prescription, contain live microorganisms in which most bacteria are similar to the beneficial bacteria that are naturally present in the human GI tract (Wilkins and Sequoia, 2017). *Lactobacillus* and *Bifidobacteria* are often used in probiotic products and have been studied in clinical trials (Kligler and Cohrssen, 2008; Raskov et al., 2016). The efficacy and safety of probiotic products for the treatment of IBS are supported by an increasing number of clinical studies. A meta-analysis (Ford et al., 2018) with 53 randomized controlled trials (RCTs) involving 5,545 patients provided data regarding the potential efficacy of probiotic combinations and specific probiotic species or strains for improving global IBS symptoms and abdominal pain. In addition to relieving symptoms, probiotics have been demonstrated to improve the quality of life (QOL) and diversify the microbial community of IBS cases in several studies (Sun et al., 2018; Preston et al., 2018).

To the best of our knowledge, although the efficacy and safety of probiotics have been confirmed by numerous studies, the best species for probiotics used in the treatment of IBS have not been identified yet (Gwee et al., 2019). Therefore, in the present study, we performed a systematic review and network meta-analysis (NMA) to compare the efficacy of probiotics for IBS to identify the best interventions.

## Methods

A systematic review and NMA were carried out in accordance with the Preferred Reporting Items for Systematic Review and Meta-analysis extension statement, including NMA (PRISMA-NMA) (Page et al., 2021).

### Search Strategy

The databases, including PubMed, Cochrane Library, Web of Science, and Medline, were searched systematically by two independent researchers on August 25, 2021, to identify RCTs exploring the efficacy of probiotics for patients with IBS. The search terms in PubMed were as follows: *(irritable bowel syndrome) OR (IBS) AND (probiotics) OR (probiotic) OR (Saccharomyces) OR (Escherichia) OR (Bifidobacterium) OR (Bacillus) OR (Lactobacillus) OR (Clostridium) AND ([randomized controlled trial{Publication Type}] OR [clinical trial{Publication Type}])*. In addition, the lists of references from the previous systematic review and meta-analysis in this field were also reviewed to identify any missing literature.

### Eligible Criteria

Studies that met the following criteria were eligible for NMA.

1. RCTs that compared the efficacy and tolerability of probiotic with placebo or another probiotic for patients with IBS were eligible.
2. The patients included in all RCTs had a well-established diagnosis of IBS, and there were no limitations on age, sex, countries, types of IBS, and the publication year of the RCTs.
3. The probiotics included the following species: *Saccharomyces boulardii* (*S. boulardii*), *Saccharomyces cerevisiae* (*S. cerevisiae*), *Escherichia coli* (*E. coli*), *Bifidobacterium bifidum* (*B. bifidum*), *Bacillus coagulans* (*B. coagulans*), *Lactobacillus acidophilus* (*L. acidophilus*), *Lactobacillus GG* (*LGG*), *Lactobacillus paracasei* (*L. paracasei*), *Lactobacillus salivarius* (*L. salivarius*), *Lactobacillus plantarum* (*L. plantarum*), *Bifidobacterium longum* (*B. longum*), *Lactobacillus casei* (*L. casei*), *Lactobacillus gasseri* (*L. gasseri*), *Bifidobacterium infantis* (*B. infantis*), *Clostridium butyricum* (*C. butyricum*), *Lactobacillus reuteri* (*L. reuteri*), and *Bifidobacterium lactis* (*B. lactis*), etc.;
4. The dosages of the probiotics and the duration of each intervention were recorded in detail.
5. The patients were required to be followed up for at least 1 week, and the studies had to report the outcome of symptom relief rate, assessment of global and individual symptom scores, QOL, and adverse events.

### Exclusion Criteria

The exclusion criteria included the following.

1. Duplicated studies and studies that were not related to our research topic were excluded.
2. Non-RCTs, observational studies, single-arm studies, case reports, reviews, meta-analyses, letters, protocols, and other such sources were excluded.
3. Papers published in a language other than English were excluded.
4. Papers without full text (or in which only the abstract was available) or the data of our target outcomes were excluded.
5. Participants with other comorbidities, such as inflammatory bowel disease, celiac disease, lactose intolerance, were excluded from the study.

### Data Extraction and Risk of Bias

Two authors independently extracted the following information from each study: author, year of publication, country, sample size, age of patients, subtypes of IBS, comparison, and treatment details (types and dosages of probiotics, response rate of placebo, duration of treatment, and outcome measures).

Two authors evaluated the risk of bias for each included RCT with the help of measures displayed in the Cochrane Handbook for Systematic Reviewers (version 5.1.0), which includes seven indicators: 1) random sequence generation (selection bias), 2) allocation concealment (selection bias), 3) blinding of patients and personnel (performance bias), 4) blinding of outcome assessment (detection bias), 5) incomplete outcome data (attrition bias), 6) selective reporting (reporting bias), and 7) other bias. Each indicator contained three levels: low risk, unclear risk, or high risk of bias.

If there were any inconsistencies or disagreements in the process of data extraction and quality assessment, the two authors discussed these issues or an independent expert in this field was consulted to reach a consensus.

### Statistical Analysis

NMA was performed using the Stata software version 15.0. For categorical data, we estimated the summary odds ratio (OR) with a 95% confidence interval (95% CI), and for continuous data, we estimated the summary standardized mean difference (SMD) with 95% CI. We showed the direct comparison between different interventions using a network diagram, in which the size of the nodes represents the sample size of each intervention, and the thickness of the continuous lines connecting the nodes indicates the number of studies directly comparing the two interventions. Subsequently, global inconsistency was evaluated, and the local inconsistency assessment was performed using the node-splitting method to check whether the estimated effects from the direct comparisons were consistent with those from the indirect comparisons. P>0.05 indicates that there were no significant differences of estimated effects between direct and indirect comparisons, thus the consistency model was used; otherwise, the inconsistency model was used. We assessed network heterogeneity across all treatment contrasts using I^2^ statistics, and loop-specific heterogeneity using the τ^2^ statistics. To rank the efficacy and safety of the interventions, we calculated the probabilities of the surface under the cumulative ranking curve (SUCRA) between all interventions for the primary and secondary outcomes. League tables containing both direct and indirect comparisons were also performed to summarize the outcomes of each indicator. Additionally, we also conducted a meta-regression analysis to explore whether the lengths and doses of interventions were associated with efficacy and adverse events of probiotics in IBS, if so, a subgroup analysis was performed.

## Results

### Study Selection and Characteristics

**Figure 1:**
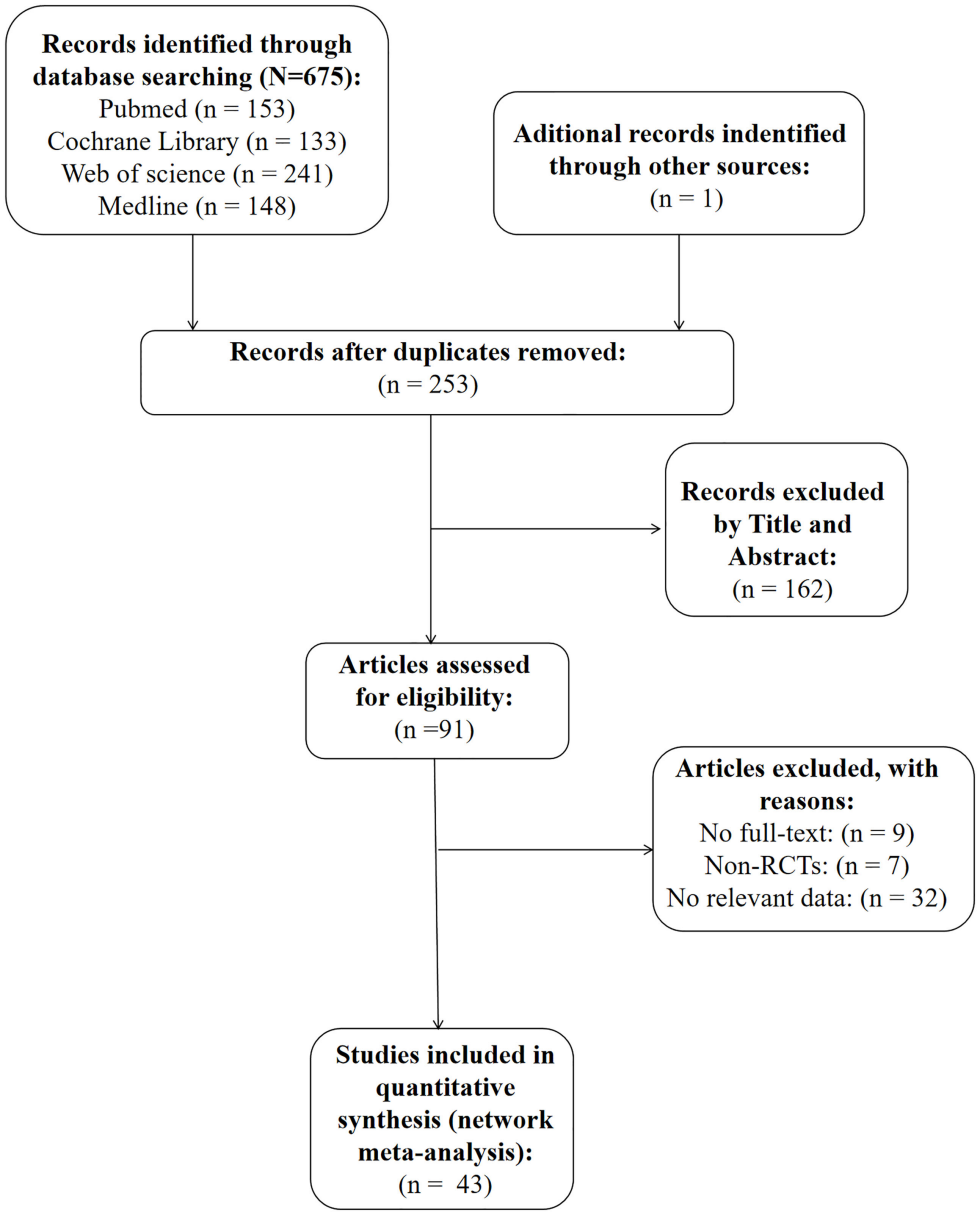
Flow diagram of assessment of studies.

As is shown in **Figure 1**, we identified a total of 676 articles in our initial search of databases and review of the lists of references. A total of 253 papers were included after accounting for the presence of duplicate papers. Furthermore, we reviewed the titles and abstracts of these papers carefully, and 162 of them were excluded because they were not relevant to our research topic. The full texts of the remaining 91 papers were further analyzed, and 48 articles were excluded (the detailed reasons for exclusion are shown in **Figure 1**). Ultimately, 43 RCTs were included in the present study.

Among the included studies, 29 RCTs (Niedzielin et al., 2001; Bauserman and Michail, 2005; Niv et al., 2005; O’Mahony et al., 2005; Whorwell et al., 2006; Sinn et al., 2008; Enck et al., 2009; Ligaarden et al., 2010; Choi et al., 2011; Guglielmetti et al., 2011; Kabir et al., 2011; Dapoigny et al., 2012; Ducrotté et al., 2012; Kruis et al., 2012; Abbas et al., 2014; Lyra et al., 2016; Majeed et al., 2016; Pineton et al., 2015; Spiller et al., 2016; Stevenson et al., 2014; Thijssen et al., 2016; Pinto-Sanchez et al., 2017; Sun et al., 2018; Sudha et al., 2018; Madempudi et al., 2019; Andresen et al., 2020; Gayathri et al., 2020; Martoni et al., 2020; Gupta and Maity, 2021) were related to 15 probiotic species, including the following species: *L. plantarum* (4 RCTs), *L. acidophilus* (4 RCTs), *B. coagulans* (4 RCTs), *S. boulardii* (3 RCTs), *S. cerevisiae* (3 RCTs), and *L. casei* (2 RCTs). One RCT (Sadrin et al., 2020), related to two different strains of *L. acidophilus*, was also identified as a RCT exploring the efficacy of a probiotic specie (*L. acidophilus*) on IBS. Unfortunately, there was only one article with respect to *B. lactis*, *L. GG*, *L. salivarius*, *B. longum*, *C. butyricum*, and *L. reuteri*.

13 RCTs (Kim et al., 2003; Kajander et al., 2005; Kim et al., 2005; Guyonnet et al., 2007; Kajander et al., 2008; Drouault-Holowacz et al., 2008; Zeng et al., 2008; Agrawal et al., 2009; Søndergaard et al., 2011; Begtrup et al., 2013; Roberts et al., 2013; Jafari et al., 2014; Wong et al., 2015) were associated with 5 types of probiotic combinations that frequently used in clinical trials as follows: 1) 1 strains of *Bifidobacterium*, 1 strains of *Lactobacillus*, and 1 strains of *Streptococcus (*hereinafter referred to as 1B1L1S*);* 2) 1 strains of *Bifidobacterium* and 2 strains of *Lactobacillus* (1B2L); 3) 1 strains of *Bifidobacterium*, 2 strains of *Lactobacillus*, and 1 strains of *Propionibacterium* (1B2L1P); 4) 1 strains of *Bifidobacterium*, 2 strains of *Lactobacillus*, and 1 strains of *Streptococcus* (1B2L1S)*;* 5) 3 strains of *Bifidobacterium*, 4 strains of *Lactobacillus*, and 1 strains of *Streptococcus* (3B4L1S). The study sample size ranged from 25 to 443, and a total of 5,531 participants were included in the NMA. The detailed patient characteristics are shown in **Table 1**.

**Table 1:** Characteristics of RCTs about the efficacy of probiotics in irritable bowel syndrome.

Study	country	Criteria used	IBS Subtypes	Intervention				Control (PLA)			^#^Outcome measures used in NMA
				Sample size	*Age	Probiotic used	Dose and duration	Sample size	*Age	Response rate	
(Abbas et al., 2014)	Pakistan	Rome III criteria	IBS-D	37	37.7 ± 11.6	*S.boulardii*	750mg/day, 6w	35	33.0 ± 12.0	N.A.	C, D, E, G
(Choi et al., 2011)	Korea	Rome II criteria	IBS-D and IBS-M	45	40.2 ± 13.1	*S.boulardii*	4x10^11^ live cells/day, 4w	45	40.6 ± 12.9	N.A.	B, C, D, E, F, G
(Kabir et al., 2011)	Bangladesh	Rome II criteria	IBS-D	35	NA	*S.boulardii*	500mg/day, 4w	35	NA	N.A.	C, D
(Gayathri et al., 2020)	India	Rome III criteria	IBS-C, IBS-D, IBS-M	52	42.25 ± 15.44	*S.cerevisiae* CNCMI-3856	4×10^9^CFU/day, 8w	48	39.6 ± 12.79	N.A.	C, G
(Pineton et al., 2015)	France	Rome III criteria	IBS-C, IBS-D, IBS-M	86	42.5 ± 12.5	*S.cerevisiae* CNCMI-3856	4×10^9^ CFU/day, 8w	93	45.4 ± 14	47%	A, C, G
(Spiller et al., 2016)	France	Rome III criteria	IBS-C	192	45.3 ± 15.7	*S.cerevisiae* CNCMI-3856	8x10^9^CFU/day, 12w	187	45.4 ± 14.1	26.90%	A, G
(Enck et al., 2009)	Germany	Kruis scale	N.A.	148	49.8(19–70)	*E.coli*	(1.5-4.5x10^7^ CFU/mL) 0.75mL drops t.i.d. for 1 week, then 1.5mL t.i.d. for weeks 2 to 8	150	49.4(18–76)	4.67%	A
(Kruis et al., 2012)	Germany	Rome II criteria	N.A.	60	46.3 ± 12.1	*E.coli* Nissle1917	(2.5-25x10^9^ CFU/capsule) o.d. for 4 days then b.d. for 12 weeks	60	45.1 ± 12.7	41.70%	A, G
(Andresen et al., 2020)	Germany	Rome III criteria	IBS-C, IBS-D, IBS-M, IBS-U	221	40·1 ± 12·8	*B.bifidum* MIMBb75	1 × 10⁹ CFU/day, 8w	222	42·6 ± 13·8	30%	A, G
(Guglielmetti et al., 2011)	Germany	Rome III criteria	IBS-C, IBS-D, IBS-M	60	36.65 ± 12.42	*B.bifidum* MIMBb75	1x10^9^ CFU/day,4w	62	40.98 ± 12.80	21%	A, G
(Gupta and Maity, 2021)	India	Rome IV criteria	N.A.	20	36.20 ± 9.81	*B.coagulans* LBSC	6 × 10⁹ CFU/day, 80d	20	34.80 ± 11.06	N.A.	C, D, E, J, K
(Majeed et al., 2016)	India	Rome III criteria	IBS-D	18	36.2 ± 11.07	*B.coagulans* MTCC5856	2×10^9^ CFU/day, 90d	18	35.4 ± 10.75	N.A.	C, D, J, K
(Madempudi et al., 2019)	India	Rome III criteria	N.A.	53	44.4	*B.coagulans* Unique IS2	2×10^9^CFU/day, 8w	55	42.3	10.91%	A, B, C, D, H, I, J, K
(Sudha et al., 2018)	India	Rome III criteria	IBS-C, IBS-D, IBS-M	72	7.86	*B.coagulans* Unique IS2	2×10^9^CFU/day, 8w	69	7.89	21.74%	A, B, C, D, E, H, I, J, K
(Lyra et al., 2016)	Finland	Rome III criteria	IBS-C, IBS-D, IBS-M, IBS-U	131	47.2 ± 12.5	*L. acidophilus* NCFM	1 × 10^10^ CFU/day, 12w	131	49.4 ± 12.9	28.40%	A, C, D, F, G
				129	47.1 ± 13.3		1 × 10⁹ CFU/day, 12w			
(Martoni et al., 2020)	India	Rome IV criteria	N.A.	111	39.41 ± 11.80	*L. acidophilus* DDS‐1	1 × 10^10^ CFU/day, 6w	109	37.61 ± 10.12	15.60%	A
				110	41.60 ± 11.11	*B.lactis* UABla‐12	1 × 10^10^ CFU/day, 6w			
(Sadrin et al., 2020)	France	Rome III criteria	N.A.	40	48.9 ± 8.4	*L.acidophilus* NCFM and *L.acidophilus* subs *p.helveticus* LAFTIL10	1×10^10^ CFU/day, 8w	40	48.9 ± 8.0	N.A.	B, C, D, G
(Sinn et al., 2008)	Korea	Rome III criteria	IBS-C, IBS-D, IBS-M	20	41.9 ± 14.4	*L.acidophilus-*SDC2012,2013	4×10^9^ CFU/day, 4w	20	47.5 ± 11.0	35%	A
(Bauserman and Michail, 2005)	USA	Rome II criteria	N.A.	25	11.6 ± 3.2	*L.GG*	2×10^10^ CFU/day, 6w	25	12.4 ± 2.9	40%	A
(O’Mahony et al., 2005)	Eire	Rome II criteria	IBS-C, IBS-D, IBS-M	26	NA	*L.salivarius* UCC4331	1 × 10^10^ CFU/day, 8w	25	NA	N.A.	B, C, D
				24	NA	*B.infantis* 35624	1 × 10^10^ CFU/day, 8w			
(Ligaarden et al., 2010)	Norway	Rome II criteria	N.A.	19	50 ± 11	*L.plantarum* MF1298	1x10^10^ CFU/day, 3w	19	50 ± 11	N.A.	B, C, D, E
(Ducrotté et al., 2012)	India	Rome III criteria	IBS-D (63.89%) and other types	108	36.53 ± 12.08	*L.plantarum* 299v(DSM 9843)	1×10^10^ CFU/day, 4w	106	38.40 ± 13.13	8.10%	A, C
(Stevenson et al., 2014)	South Africa	Rome II criteria	IBS-C, IBS-D	54	48.15 ± 13.48	*L.plantarum 299v(DSM 9843)*	1×10^10^ CFU/day, 8w	27	47.27 ± 12.15	N.A.	B, F
(Niedzielin et al., 2001)	Poland	Clinical diagnosis	N.A.	20	48 ± 18	*L.plantarum 299v(DSM 9843)*	2× 10^10^ CFU/day, 4w	20	42 ± 15	15%	A
(Pinto-Sanchez et al., 2017)	Canada	Rome III criteria	IBS-D, IBS-M	22	46.5 (30-58)	*B.longum* NCC3001	1x10^10^ CFU/day, 6w	22	40.0 (26-57)	35%	A, B, G
(Dapoigny et al., 2012)	France	Rome III criteria	IBS-C, IBS-D, IBS-M, IBS-U	25	48.0 ± 10.8	*Lactobacillus casei rhamnosus* LCR35	6x10^8^ CFU/day, 4w	25	48.0 ± 10.8	40%	A
(Thijssen et al., 2016)	Netherlands	Rome II criteria	IBS-C, IBS-D, IBS-M, IBS-U	39	41.1 ± 14.8	*L.casei* iShirota	1.3×10^10^ CFU/day, 8w	41	42.4 ± 13.5	29%	A
(Whorwell et al., 2006)	UK	Rome II criteria	IBS-C, IBS-D, IBS-M, IBS-U	90	40.8 ± 10.44	*B.infantis*35624	1×10^6^ CFU/day, 4w	92	42.4 ± 10.45	About 40%	A, B, C, D, E
				90	42.7 ± 10.44		1×10^8^ CFU/day, 4w			
				90	41.8 ± 10.44		1×10^10^ CFU/day, 4w			
(Sun et al., 2018)	China	Rome III criteria	IBS-D	105	43.00 ± 12.45	*C.butyricum*	5.67× 10^7^ CFU/day, 4w	95	44.91 ± 13.01	35%	A, B, C, D, F, G
(Niv et al., 2005)	Israel	Rome II criteria	IBS-C, IBS-D, IBS-M	27	45.7 ± 14.2	*L.reuteri* ATCC55730	2×10^8^ CFU/day, 6m	27	45.6 ± 16.1	N.A.	B, G
(Agrawal et al., 2009)	UK	Rome III criteria	IBS-C	17	42(24,69)	*B. lacti* DN-173010*, S.thermophilus, and L. bulgaricus*	7.35×10^10^ CFU/day, 4w	17	37(20,59)	N.A.	J, K
(Begtrup et al., 2013)	Denmark	Rome III criteria	IBS-C, IBS-D, IBS-M, IBS-U	67	31.63 ± 10.05	*L. paracasei ssp paracasei* F19*, L. acidophilus* La5*, and B. lactis* Bb12	5.2x10^10^ CFU/day, 6m	64	29.38 ± 8.64	29%	H, I, J, K
(Søndergaard et al., 2011)	Denmark and Sweden	Rome II criteria	N.A.	27	53.9(29–67)	*L.paracasei ssp paracasei* F19*, L.acidophilus* La5*, and B.lactis* Bb12	2.5x10^10^ CFU/day, 8w	25	48.5(29–67)	About 25%	H, K
Guyonnet 2007 (Guyonnet et al., 2007)	France	Rome II criteria	N.A.	135	49.4 ± 11.4	*B.animalis* DN173 010*, S.thermophilus, and L.bulgaricus*	2.98× 10^10^ CFU/day,6w	132	49.2 ± 11.4	56.80%	H, I, J, K
(Drouault-Holowacz et al., 2008)	France	Rome II criteria	IBS-C, IBS-D, IBS-M	48	47 ± 14	*B. longum* LA 101*, L. acidophilus* LA 102*, L. lactis* LA 103*, and S. thermophilus* LA 104	1×10^10^ CFU/day, 4w	52	44 ± 14	42.30%	H, J
(Jafari et al., 2014)	Iran	Rome III criteria	N.A.	54	36.6 ± 12.1	*B.animalis subsp. lactis* BB-12^®^ *, L.acidophilus LA-5^®^, L.delbrueckii subsp. bulgaricus* LBY-27*, S.thermophilus* STY-31	8× 10⁹ CFU CFU/day, 4w	54	36.6 ± 12.1	47%	H
(Kim et al., 2003)	USA	Rome II criteria	IBS-D	12	48 ± 5.7	VSL#3: (Threes trains of *Bifidobacterium* (*B.longum, B.infantis and B.breve)*; four strains of *Lactobacillus* (*L.acidophilus, L.casei, L.bulgaricus* and *L.plantarum*); and one strain of *Streptococcus* (*S.salivarius subspecies thermophilus*)	4.5×10^11^ bacteria/day, 8w	13	38 ± 3.4	38%	H, I, J, K
(Zeng et al., 2008)	China	Rome II criteria	IBS-D	14	44.6 ± 12.4	*S.thermophilus, L.bulgaricus, L.acidophilus, and B.longum*	2.6×10^10^ CFU/day, 4w	15	45.8 ± 9.2	N.A.	I, J, K
(Kajander et al., 2005)	Finland	Rome I and II criteria	IBS-C, IBS-D, IBS-M	52	46(23–65)	*L.rhamnosus* GG*, L.rhamnosus* Lc705*, P.freudenreichii, and B.breve* Bb99	8–9×10^9^/CFU/day, 6m	51	45(21–65)	33.33%	H, J, K
(Kajander et al., 2008)	Finland	Rome II criteria	IBS-C, IBS-D, IBS-M	43	50 ± 13	*L.rhamnosus GG, L.rhamnosus Lc705 DSM 7061, P.freudenreichii, and B.animalis*	4.8×10^9^ CFU/day, 20w	43	46 ± 13	N.A.	I
(Kim et al., 2005)	USA	Rome II criteria	N.A.	24	40 ± 14.70	VSL#3: Threes trains of *Bifidobacterium* (*B.longum, B.infantis and B.breve)*; four strains of *Lactobacillus* (*L.acidophilus, L.casei, L.bulgaricus* and *L.plantarum*); and one strain of *Streptococcus* (*S.salivarius subspecies thermophilus*)	9×10^11^ CFU/day; 8w	24	46 ± 14.70	33%	H, J, K
(Roberts et al., 2013)	England	Rome III criteria	IBS-C, IBS-M	88	44.66 ± 11.98	*B.lactis I-2494, S.thermophilus, and L.bulgaricus*	2.98×10^10^ CFU/day, 12w	91	43.71 ± 12.76	68.30%	H
(Wong et al., 2015)	Singapore	Rome III criteria	N.A.	20	53.35 ± 18.56	VSL#3: Threes trains of *Bifidobacterium* (*B.longum, B.infantis and B.breve)*; four strains of *Lactobacillus* (*L.acidophilus, L.casei, L.bulgaricus* and *L.plantarum*); and one strain of *Streptococcus* (*S.salivarius subspecies thermophilus*)	9× 10^11^ CFU/day, 6w	22	40.86 ± 16.46	N.A.	J, K

^*^Mean ± sd or mean (range).

A: Symptom relief rate; B: global symptom scores; C: abdominal pain scores; D: bloating scores; E: straining scores; F: QOL; G: AEs; and H-K refers to the outcome relevant to the comparisons of B. coagulans with different probiotic combinations: H: Symptom relief rate; I: global symptom scores; J:abdominal pain scores; K: bloating scores.

N.A., Not reported or not available.

### Risk of Bias

The risk of bias assessment for all the included RCTs is presented in **Supplementary Figure 1** (**Figure S1**). Overall, twelve trials were judged to have a low risk of bias across all domains. Two trials were judged to have a high risk of bias for blinding of the outcome assessment. Almost all the RCTs were judged to have a low risk of bias for allocation concealment, blinding of participants and personnel, incomplete outcome data, and selective reporting, except for two trials that had unclear risk in the domain of allocation concealment, one trial that had an unclear risk for blinding of participants and personnel, and one trial that had an unclear risk for incomplete outcome data.

### Primary Outcomes: Effect of Different Probiotic Species on Symptom Relief Rate

**Figure 2:**
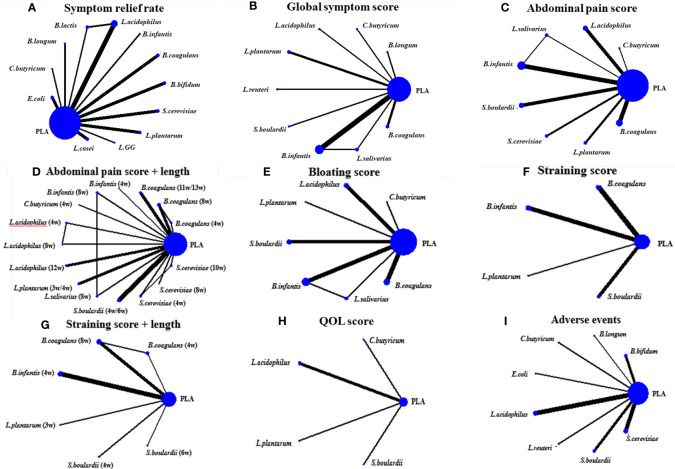
The network plots. **(A)** was the network plot about the effect of probiotics on improving the symptom relief rate of IBS patients; **(B)** Global symptom score; **(C)** Abdominal pain score; **(D)** Bloating score; **(E)** Straining score; **(F)** QOL score; **(G)** Adverse events; **(H)** Subgroup analysis on treatment length of probiotics for improving the abdominal pain of IBS patients; **(I)** Subgroup analysis on treatment length of probiotics for improving the straining scores of IBS patients.

A total of 19 RCTs explored the efficacy of probiotics on symptom relief rate, and the network plots are presented in **Figure 2A**. The results of the global and local inconsistency tests are presented in **Figure S2 **and** Table S1**. Both tests showed that there was no significant inconsistency between the direct comparisons and indirect comparisons; thus, the consistency model was used. The NMA revealed that *B. coagulans* (OR 60.73, 95% CI, 14.83 to 248.61), *L. plantarum* (OR 15.62, 95% CI 2.90 to 84.21), and *L. acidophilus* (OR 3.00, 95% CI 1.03 to 8.68) had a greater effect on symptom relief rate in patients with IBS compared with placebo (PLA). The SUCRA analysis (**Table S2 **and** Figure S3**) and league table (**Table 2**) showed that *B. coagulans* had the best rank among all the treatment interventions; meanwhile, *L. plantarum* ranked second, *L. acidophilus* ranked third, and PLA ranked last.

**Table 2:** Odds ratio (OR) with 95% confidence interval on symptom relief rate.

B.coagulans												
3.89 (0.43,34.97)	**L.plantarum**											
**20.27 (3.47,118.40)**	5.21 (0.72,37.92)	**L.acidophilus**										
**23.13 (3.57,149.71)**	5.95 (0.74,47.62)	1.14 (0.23,5.76)	**B.bifidum**									
**24.02 (2.04,283.25)**	6.18 (0.44,86.09)	1.19 (0.12,11.67)	1.04 (0.10,11.08)	**B.longum**								
**26.05 (3.86,175.58)**	6.70 (0.80,55.75)	1.29 (0.24,6.81)	1.13 (0.19,6.66)	1.08 (0.10,11.94)	**E.coli**							
**32.93 (3.56,304.61)**	8.47 (0.76,94.16)	1.62 (0.21,12.29)	1.42 (0.17,11.78)	1.37 (0.10,19.55)	1.26 (0.15,10.84)	**C.butyricum**						
**40.65 (4.83,341.86)**	**10.45 (1.03,106.03)**	2.01 (0.41,9.74)	1.76 (0.24,13.13)	1.69 (0.13,22.30)	1.56 (0.20,12.12)	1.23 (0.12,12.92)	**B.lactis**					
**43.74 (6.87,278.61)**	**11.25 (1.42,89.04)**	2.16 (0.43,10.73)	1.89 (0.34,10.52)	1.82 (0.17,19.17)	1.68 (0.29,9.76)	1.33 (0.16,10.83)	1.08 (0.15,7.93)	**S.cerevisiae**				
**49.97 (5.53,451.25)**	**12.85 (1.18,139.74)**	2.47 (0.33,18.16)	2.16 (0.27,17.43)	2.08 (0.15,29.08)	1.92 (0.23,16.04)	1.52 (0.14,16.93)	1.23 (0.12,12.57)	1.14 (0.14,9.08)	**B.infantis**			
**51.53 (4.56,581.63)**	13.25 (0.99,177.27)	2.54 (0.27,23.89)	2.23 (0.22,22.71)	2.14 (0.13,36.21)	1.98 (0.19,20.83)	1.56 (0.11,21.44)	1.27 (0.10,16.03)	1.18 (0.12,11.85)	1.03 (0.08,13.84)	**L.GG**		
**66.15 (9.28,471.33)**	**17.01 (1.94,149.18)**	3.26 (0.58,18.48)	2.86 (0.46,17.96)	2.75 (0.24,31.69)	2.54 (0.39,16.60)	2.01 (0.22,18.09)	1.63 (0.20,13.33)	1.51 (0.25,9.33)	1.32 (0.15,11.64)	1.28 (0.12,14.14)	**L.casei**	
**60.73 (14.83,248.61)**	**15.62 (2.90,84.21)**	**3.00 (1.03,8.68)**	2.63 (0.77,8.95)	2.53 (0.33,19.15)	2.33 (0.64,8.44)	1.84 (0.33,10.31)	1.49 (0.30,7.37)	1.39 (0.42,4.61)	1.22 (0.22,6.59)	1.18 (0.16,8.47)	0.92 (0.23,3.60)	**PLA**

Lower left triangle refers to the OR from the network meta-analysis. (e.g.,the OR [95%CI] of symptom relief rate between B.coagulans and placebo is 60.73[14.83-248.61]). The data in bold indicates that the effect size is statistically significant (P < 0.05).

**Figure 3:**
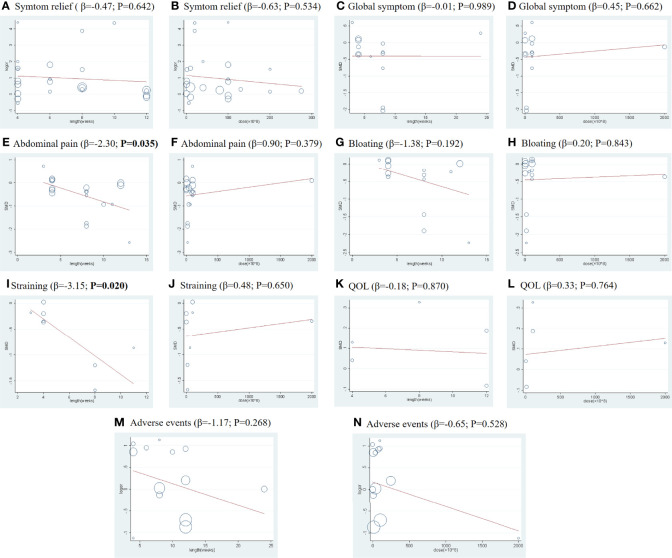
Meta-regression by treatment lengths and doses for all primary and secondary outcomes: **(A, C, E, G, I, K, M)** indicate the meta-regression analysis by treatment lengths for the outcomes of symptom relief rate, global symptom score, abdominal pain score, bloating score, straining scores, QOL, and adverse events, respectively; **(B, D, F, H, J, L, N)** indicate the meta-regression analysis by doses for the outcomes of symptom relief rate, global symptom score, abdominal pain score, bloating score, straining scores, QOL, and adverse events, respectively. The abscissa (X) represents the duration (weeks) or dose (×108), and the ordinate (Y) represents the SMD.

Significant heterogeneity was observed across all treatment contrasts (I^2^ = 85.5%), but no evidence of loop-specific heterogeneity was found (τ^2 =^ 0). The meta-regression analysis by treatment dose and length did not significantly influence the SMD estimates for this outcome (**Figures 3A, B**).

### Primary Outcomes: Effect of Different Probiotic Species on Global Symptom Scores

A total of 13 RCTs comparing the effect of probiotics on the global symptom scores of patients with IBS were included. The network plot is shown in **Figure 2B**. Both the global and local inconsistency tests revealed a significant inconsistency between direct and indirect comparisons (**Figure S4** and **Table S3**), which indicated that the inconsistency model should be used. The result of NMA revealed a significant improvement in the global symptom scores in patients who received *B. coagulans* (SMD −1.99, 95% CI −2.39 to −1.59) and *Bifidobacterium infantis* (SMD −0.74, 95% CI −1.47 to −0.01) compared with those who received PLA. Based on the SUCRA analysis (**Table S4** and **Figure S5**) and league table (**Table 3**), *B. coagulans*, *C. butyricum*, and *Bifidobacterium longum* ranked as the top three interventions in improving the global symptom scores of patients with IBS, while *L. plantarum* ranked last.

**Table 3:** Standardized mean differences (SMDs) and 95% CI on global symptom scores.

B.coagulans									
**-1.65 (-2.37,-0.93)**	**C.butyricum**								
**-1.59 (-2.63,-0.55)**	0.06 (-1.07,1.19)	**B.longum**							
**-1.67 (-2.37,-0.97)**	-0.02 (-0.84,0.81)	-0.08 (-1.20,1.04)	**L.acidophilus**						
**-1.70 (-2.47,-0.92)**	-0.05 (-0.94,0.84)	-0.11 (-1.28,1.06)	-0.03 (-0.91,0.85)	**L.salivarius**					
**-1.86 (-2.55,-1.18)**	-0.21 (-1.03,0.60)	-0.27 (-1.38,0.84)	-0.20 (-0.99,0.60)	-0.17 (-1.03,0.70)	**S.boulardii**				
**-1.94 (-2.42,-1.45)**	-0.29 (-0.94,0.36)	-0.35 (-1.35,0.65)	-0.27 (-0.91,0.37)	-0.24 (-0.96,0.47)	-0.07 (-0.69,0.54)	**B.infantis**			
**-1.99 (-2.39,-1.59)**	-0.40 (-1.36,0.57)	-0.05 (-0.32,0.22)	-0.34 (-0.93,0.26)	0.28 (-0.45,1.01)	-0.29 (-0.95,0.37)	**-0.74 (-1.47,-0.01)**	**PLA**		
**-2.27 (-3.10,-1.43)**	-0.62 (-1.56,0.32)	-0.68 (-1.89,0.53)	-0.60 (-1.53,0.33)	-0.57 (-1.56,0.41)	-0.41 (-1.32,0.51)	-0.33 (-1.11,0.45)	-0.21 (-0.68,0.25)	**L.reuteri**	
**-2.20 (-2.82,-1.58)**	-0.55 (-1.31,0.20)	-0.61 (-1.68,0.46)	-0.53 (-1.27,0.21)	-0.50 (-1.31,0.31)	-0.34 (-1.06,0.39)	-0.26 (-0.80,0.28)	0.32 (-0.25,0.89)	0.07 (-0.80,0.94)	**L.plantarum**

Lower left triangle refers to the SMD from the network meta-analysis. (eg.,the SMD [95%CI] of global symptom scores between B.coagulans and placebo is -1.99[-2.39, -1.59]). The data in bold indicates that the effect size is statistically significant (P<0.05).

In this outcome, we found obvious heterogeneity across all treatment contrasts (I^2^ = 91.2%), but no heterogeneity (τ^2 =^ 0) in the loop of NMA. Meta-regression analysis showed that treatment dose and length did not significantly influence the SMD estimates for global symptom scores (**Figures 3C, D**).

### Primary Outcomes: Effect of Different Probiotic Species on Abdominal Pain Scores

A total of 16 RCTs reported the effect of probiotics on abdominal pain scores in patients with IBS, and the network diagram is shown in **Figure 2C**. Both the global inconsistency test (**Figure S6**) and node-splitting assessment (**Table S5**) showed no significant inconsistency between direct and indirect comparisons; therefore, the consistency model was used. The results of NMA (**Table 4**) revealed a significant improvement in the abdominal pain scores in patients who received *B. coagulans* (SMD −1.71, 95% CI −2.15 to −1.27) and *S. cerevisiae* (SMD −0.54, 95% CI −1.08 and −0.00) than those who received PLA. The SUCRA analysis (**Table S6** and **Figure S7**) demonstrated that *B. coagulans* ranked first in improving the abdominal pain scores of patients with IBS, while *S. cerevisiae* ranked second, *C. butyricum* ranked third, and *S. boulardii* ranked last.

**Table 4:** Standardized mean differences (SMDs) and 95% CI on abdominal pain scores.

B.coagulans								
**-1.17 (-1.87,-0.47)**	**S.cerevisiae**							
**-1.45 (-2.31,-0.60)**	-0.29 (-1.19,0.62)	**C.butyricum**						
**-1.52 (-2.42,-0.63)**	-0.35 (-1.30,0.59)	-0.07 (-1.13,1.00)	**L.salivarius**					
**-1.56 (-2.18,-0.94)**	-0.39 (-1.08,0.30)	-0.11 (-0.95,0.74)	-0.04 (-0.93,0.85)	**L.acidophilus**				
**-1.60 (-2.18,-1.01)**	-0.43 (-1.09,0.23)	-0.14 (-0.97,0.68)	-0.07 (-0.85,0.70)	-0.03 (-0.61,0.54)	**B.infantis**			
**-1.71 (-2.45,-0.97)**	-0.54 (-1.34,0.26)	-0.25 (-1.19,0.69)	-0.19 (-1.16,0.79)	-0.15 (-0.88,0.59)	-0.11 (-0.82,0.60)	**L.plantarum**		
**-1.71 (-2.15,-1.27)**	**-0.54 (-1.08,-0.00)**	-0.26 (-0.99,0.47)	-0.19 (-0.97,0.59)	-0.15 (-0.58,0.28)	-0.12 (-0.50,0.27)	-0.00 (-0.59,0.59)	**PLA**	
**-2.03 (-2.68,-1.39)**	**-0.86 (-1.58,-0.15)**	-0.58 (-1.45,0.29)	-0.51 (-1.42,0.40)	-0.47 (-1.11,0.17)	-0.44 (-1.04,0.17)	-0.33 (-1.08,0.43)	-0.32 (-0.79,0.15)	**S.boulardii**

Lower left triangle refers to the SMD from the network meta-analysis. The data in bold indicates that the effect size is statistically significant (P<0.05).

There was a significant heterogeneity across all treatment contrasts (I^2^ = 90.4%), but no loop-specific heterogeneity (τ^2 =^ 0) in this outcome. The meta-regression analysis (**Figures 3E, F**) showed that treatment duration, as a confounder, can significantly influence the efficacy of probiotics in improving the symptoms of abdominal pain (Coef = −2.30; p= 0.035) in patients with IBS.

Subsequently, we performed a subgroup analysis of treatment duration (**Figure 2D**). No evidence of loop-specific heterogeneity was found (τ^2 =^ 0). The consistency model was used based on the results of the global inconsistency test (**Figure S8**) and node-splitting assessment (**Table S7**), both of which showed that there was no significant inconsistency between the direct and indirect comparisons. The results of the NMA indicated that the patients who received *B. coagulans* (8 w) (SMD −2.13, 95% CI −2.84 to −1.41), *B. coagulans* (11 w/13 w) (SMD −1.61, 95% CI −2.46 to −0.76), and *S. cerevisiae* (10 w) (SMD −1.00, 95% CI −2.00 to −0.00) had lower abdominal pain scores than those who received PLA. Based on the results of the SUCRA (**Table S8** and **Figure S9**) and league table (**Table S9**), we found that *B. coagulans* (8 w), *B. coagulans* (11 w/13 w), and *S. cerevisiae* (10 w) ranked as the top three among all the interventions, while *S. boulardii* (4 w/6 w) ranked last.

### Primary Outcomes: Effect of Different Probiotic Species on Bloating Scores

The effect of probiotics on abdominal bloating was reported in 13 RCTs, and the network plot is presented in **Figure 2E**. The consistency model was used as there was no significant inconsistency found in both the global and local inconsistency tests (**Table S10** and **Figure S10**). Only patients who received *B. coagulans* (SMD −1.42, 95% CI −1.87 to −0.96) had a significant improvement in abdominal bloating scores compared with those who received PLA. The SUCRA analysis (**Table S11** and **Figure S11**) and league table (**Table S12**) indicated that *B. coagulans* ranked best among all the other interventions, while *B. infantis* ranked second, *L. acidophilus* ranked third, and *L. plantarum* ranked last.

In this primary outcome, the heterogeneity across all treatment contrasts was significant (I^2^ = 90.0%), which was in contrast to the loop-specific heterogeneity (τ^2 =^ 0). Additionally, the meta-regression analysis using treatment length and dose did not influence the SMD estimates for bloating sores significantly (**Figures 3G, H**).

### Primary Outcomes: Effect of Different Probiotic Species on Straining Scores

There were only seven RCTs involved when comparing the effect of probiotics on straining scores. The network plot is shown in **Figure 2F**. Due to a lack of inconsistency resources, the consistency model was used. The results of the NMA showed that the patients who were administered *B. coagulans* (SMD −1.29, 95% CI −1.63 to −0.94) had lower straining scores than those who were administered PLA. Based on the SUCRA analysis in **Table S12** and **Figure S13** and the league table in **Table S14**, *B. coagulans* had the best rank among all other interventions in improving straining scores of patients with IBS, followed by *B. infantis* and *L. plantarum*; meanwhile, PLA ranked last.

The heterogeneity was significant across all treatment contrasts (I^2^ = 88.7%) and the meta-regression analysis (**Figures 3I, J**) showed that treatment duration can significantly influence the efficacy of probiotics in improving the symptoms of straining (Coef = −3.15; p = 0.020) in patients with IBS. Therefore, we performed a subgroup analysis of treatment lengths (**Figure 2G**). Both the global and local inconsistency tests showed a significant inconsistency (**Table S13** and **Figure S15**), which indicated that the inconsistency model should be used. The NMA results revealed a significant improvement in symptoms of straining in the patients who received *B. coagulans* (8 w) (SMD −1.60, 95% CI −2.12 to −1.08) compared to those who received PLA. Additionally, the SUCRA (**Table S16** and **Figure S14**) and league table (**Table S17**) showed that *B. coagulans* (8 w) ranked first, *S. boulardii* (4 *w*) ranked second, *B. coagulans* (4 w) ranked third, while *S. boulardii* (6 w) ranked last.

### Secondary Outcomes: Effect of Different Probiotic Species on QOL

The effect of probiotics on the QOL of patients with IBS was only reported in four RCTs. The network plot is shown in **Figure 2H**. The consistency model was used due to the lack of inconsistent resources. The NMA results showed that there are no treatment interventions better than PLA in improving the QOL of patients with IBS. The results of the SUCRA analysis, available in **Table S18, 19** and **Figure S15**, showed that *L. plantarum* ranked first in improving the QOL of patients with IBS; meanwhile, *S. boulardii* ranked second, *L. acidophilus* ranked third, and PLA ranked last. No significant heterogeneity was observed across all treatment contrasts (I^2^ = 0.0%). Meta-regression by treatment length and dose did not significantly influence the SMD estimates for QOL (**Figures 3K, L**).

### Secondary Outcomes: Adverse Events (AEs)

Total AEs were reported in 13 RCTs, and the network plot is presented in **Figure 2I**. The consistency model was used due to a lack of inconsistent resources. The NMA results revealed that only patients who received *L. acidophilus* had a lower incidence of AEs compared with patients who received PLA (OR 0.47, 95% CI 0.32, 0.67). Based on the SUCRA analysis (**Table S20 and Figure S16**) and league table (**Table S21**), *L. acidophilus* ranked first among all the other interventions, PLA ranked second, *L. reuteri* ranked third, and *C. butyricum* ranked last. Additionally, we observed significant heterogeneity (I^2^ = 59.3%) across all studies in this outcome. Meta-regression by treatment duration and dose did not significantly influence the SMD estimates for the incidence of AEs (**Figures 3M, N**).

### Comparisons of *B. coagulans* With Different Probotic Combinations for the Treatment of IBS

The evidences above revealed that *B. coagulans* was more effective in improving several IBS related symptoms than other probiotic species, thus, we further explored its efficacy compared to different types of probiotic combinations. Interestingly, based on the results from SUCRA analysis (**Figures S17-20** and **Tables S22-25**), we found that *B. coagulans* had the best rank among all the probiotic combinations in improving symptom relief rate, as well as global symptom, abdominal pain, and bloating scores. Simultaneously, the probiotic combinations 1B2L1S (with 1 strains of *Bifidobacterium*, 2 strains of *Lactobacillus*, and 1 strains of *Streptococcus*) ranked second in improving global symptom and abdominal pain scores.

### Comparisons of Different Strains of *B. coagulans* for the Treatment of IBS

As for the symptom relief rate and global symptom scores, only *B. coagulans* Unique IS2 was involved, thus it is not difficult to conclude that *B. coagulans* Unique IS2 ranked first in improving the symptom relief rate and global symptoms of IBS relatively among all interventions. In terms of the ability to alleviate abdominal pain of IBS patients, the league table (**Table S26**) showed that *B.coagulans* MTCC5856 ranked first and *B.coagulans* Unique IS2 ranked second, which was consistent with the result of abdominal bloating scores (**Table S27**). Lastly, *B.coagulans* Unique IS2 also exhibited the highest probability to be the optimal strains in improving the symptom of straining (**Table S28**).

## Discussion

To date, the guidelines on the treatment of IBS with probiotics remain controversial. The British Society of Gastroenterology guidelines (Vasant et al., 2021) on the management of IBS, which was updated in 2021, reported that probiotics may be an effective treatment for improving global symptoms and abdominal pain in patients with IBS, which was consistent with the recommendations of the Canadian Association of Gastroenterology (Moayyedi et al., 2019) and the Japanese Society of Gastroenterology (Fukudo et al., 2021). In contrast, the guidelines from the American College of Gastroenterology (Lacy et al., 2021) suggest against the use of probiotics for the treatment of global IBS symptoms. Despite the controversies among different clinical practice guidelines, the effectiveness of probiotics in treating patients with IBS has not been completely validated before (Gwee et al., 2019) due to significant heterogeneity, publication bias, and inconsistent results in some meta-analyses, as well as several small sample size RCTs without rigorous endpoints based on US Food and Drug Administration (USFDA), and multiple types of probiotics without adequate validations, which may also contribute to the low level of evidence in the guidelines (Lacy et al., 2021).

To the best of our knowledge, this is the first study to simultaneously compare the efficacy of different probiotic species used for the treatment of IBS. The strength of this systematic review and NMA is that we performed a meta-regression analysis on the duration and doses of different treatments to explore whether these factors influence the outcomes, and if there were any influences, a subgroup analysis was conducted, adding rigor to our results. The main findings of our NMA were that *B. coagulans* was effective in increasing the symptom relief rate of patients with IBS, as well as in improving global symptoms, abdominal pain, bloating, and straining. Moreover, the meta-regression on treatment duration can significantly influence the SMD estimates of abdominal pain and straining scores, which indicates that increased treatment duration was a factor that negatively influenced both outcomes. The subgroup analysis of treatment durations indicated that the administration of *B. coagulans* for 8 weeks increased the effectiveness in the improvement of symptoms of abdominal pain and straining in patients with IBS. Unfortunately, due to insufficient original data of the included RCTs, NMA could not be performed to determine its efficacy in improving the QOL of patients with IBS and the AEs associated with this disease. Additionally, *B. coagulans* still had significant effects in improving symptom relief rate, as well as global symptom, abdominal pain, and bloating scores compared to different types of probiotic combinations in present study, which further validated the pronounced efficacy of *B. coagulans*.

*B. coagulans* is a spore-forming bacteria widely used in commercial probiotic formulations owing to its outstanding properties which are partly associated with its encapsulated coating that can protect it from drought conditions and allow it to survive and proliferate in various secretions of the GI tract, such as gastric acid, pepsin, pancreatin, digestive enzymes, and bile (Mu and Cong, 2019). Additionally, it can produce a range of proteins, antimicrobial substances, and vitamins, as well as modulate the gut microbiome, strengthen the body’s immunity (Elshaghabee et al., 2017; Maity et al., 2020), and treat various ailments such as *Helicobacter pylori* infection, gingivitis, and IBD. Although there are only a few RCTs regarding the use of different strains of *B. coagulans* for patients with IBS, their efficacy and safety are apparent. In two different studies, Madempudi et al. (Sudha et al., 2018; Madempudi et al., 2019) demonstrated that *B. coagulans* Unique IS2 was effective in relieving IBS-associated symptoms, such as abdominal pain, bloating, urgency, and straining, in improving stool consistency, and in increasing the serum anti-inflammatory factor IL-10 in children and adults with acceptable tolerability. Majeed et al. (2016) and Gupta et al. (Gupta and Maity, 2021) found that *B. coagulans* can improve the QOL of patients with IBS-D and significantly relieve the symptoms of diarrhea and constipation in the patients. To the best of our knowledge, our study is the first to compare the effectiveness of *B. coagulans* with other interventions and confirms the significant efficacy of *B. coagulans* in patients with IBS, especially at 8 weeks. Nevertheless, it is valuable to note that the benefits provided by probiotics are strain-specific rather than species-specific and genus-specific (Majeed et al., 2016); therefore, the health benefits may vary based on different strains of *B. coagulans*. Thus, we compared the efficacy of different strains of *B. coagulans* for the treatment of IBS, which revealed that *B. coagulans* Unique IS2 exhibited the highest probability to be the optimal strains in improving symptom relief rate, global symptom scores, and the symptom of straining. Meanwhile, *B.coagulans* MTCC5856 ranked first in alleviating abdominal pain and abdominal bloating.

Increasing evidence, including the biopsychosocial model of IBS, suggests that in patients with IBS, psychosocial factors (anxiety, stress) can be secondary to abdominal symptoms (bottom-up); in turn, intestinal (physiological) functions, such as visceral sensitivity, motility, and stress reactivity of the gut can be impacted by psychosocial factors (top-down) (Fond et al., 2014). It is believed that both the gut microbiome and the gut-brain axis play an important role in the bidirectional signaling between the brain and the gut (Schmidt, 2015) through the neurological, endocrine, and immune pathways (Carabotti et al., 2015), especially *via* the former two pathways (Ng et al., 2018). It has also been reported that the gut microbiome has a direct influence on stress reactivity by stimulating the vagus nerve and the enteric nervous system (Ng et al., 2018), as well as by synthesizing and modulating neurotransmitters (Yano et al., 2015). Thus, in IBS cases, the disturbed QOL attributed to comorbidity of abdominal symptoms, extra-intestinal symptoms, and psychiatric symptoms (Creed et al., 2001; Spiegel et al., 2004) can be improved by alleviating IBS-related pain (abdominal symptoms) (El-Serag and Olden, 2002) by regulating the gut microbiome with probiotic therapies.

Interestingly, a previous study found that some probiotics, such as *Lactobacillus acidophilus* NCFM, can modify the expression of pain-associated receptors, such as μ-opioid and cannabinoid receptors, in the GI tract in mice and humans (Rousseaux et al., 2007; Ringel-Kulka et al., 2014), thereby improving the symptoms of abdominal pain. Some bacterial species, such as *Enterobacteriaceae* and *Clostridia*, are more prone to producing intestinal gas and generating abnormal patterns of short-chain fatty acids than others; thus, the imbalance in gut microbiota may exacerbate the symptoms of bloating (King et al., 1998; O’Sullivan and O’Morain, 2000). The modification of microbiota attributed to probiotics may improve bloating symptoms by decreasing the production of intestinal gas and promoting gut motility. Despite the presence of ample data regarding this issue, the precise mechanism of action of specific probiotic species or strains in improving the symptoms of IBS is still speculative and remains to be confirmed.

It is notable that the efficacy of probiotic combinations are not necessarily better than mono-strain probiotics in present study, which was consistent with the outcomes of a research performed by Ringel-Kulka et al. (2014). Due to the different probiotic combinations used in many studies, it is difficult for us to determine which probiotic combination is more effective for IBS patients. Therefore, multi-center clinical trials with large sample sizes are still needed. Moreover, incorporating *B. coagulans* into a probiotic combination, or genetically engineering it to amplify its biological function may be a future research target to treat IBS patients.

Our NMA has several limitations. First, although we investigated all RCTs with synthesizable data, a lack of available trials or trials with large sample sizes for direct comparisons remains, which may have influenced our results. Second, due to the limited original data, we were unable to evaluate more clinical indicators, such as bowel habits, stool consistency, gut motility, serum inflammation-related factors, and the gut microbiome. Third, the methodologies of included RCTs vary in design, population, diagnosis criteria, IBS subtypes, and durations, and the outcome measures were different, making it difficult to draw robust conclusions. Therefore, the results of this NMA should be interpreted with caution.

## Conclusions

The findings of our NMA suggest that *B. coagulans* was particularly effective in improving symptom relief rate, as well as global symptoms, abdominal pain, bloating, and straining scores. Furthermore, patients with IBS who received *L. acidophilus* had a lower incidence of AEs than those who received other treatments. Although some of the included RCTs are underpowered due to limited number of cases and different outcome measures, the results of our study may be useful in establishing treatment guidelines for IBS using probiotics, considering that there are only a few reports in the literature that have made direct comparisons between individual therapies for IBS.

## Author Contributions

Guarantor of the article: LD is guarantor. Author contributions: TZ, CZ, JZ, FS, and LD conceived and drafted the study. CZ screened abstracts, TZ collected all data. TZ, CZ, JZ, and FS analyzed and interpreted the data. TZ and CZ drafted the manuscript. LD acquired the funding and performed critical revisions of the manuscript. All authors have approved the final draft of the manuscript.

## Funding

This study was funded by and National Key R&D Program of China (2019YFA0905604) and National Natural Science Foundation of China (82170557).

## Conflict of Interest

The authors declare that the research was conducted in the absence of any commercial or financial relationships that could be construed as a potential conflict of interest.

## Publisher’s Note

All claims expressed in this article are solely those of the authors and do not necessarily represent those of their affiliated organizations, or those of the publisher, the editors and the reviewers. Any product that may be evaluated in this article, or claim that may be made by its manufacturer, is not guaranteed or endorsed by the publisher.

## Abbreviations

IBS, Irritable bowel syndrome; GI, gastrointestinal; IBS-D, diarrhea predominant IBS; RCTs, randomized controlled trials; QOL, quality of life; NMA, network meta-analysis; OR, odds ratio; 95% CI, 95% confidence interval; SMD, standardized mean difference; SUCRA, surface under the cumulative ranking curve; AEs, adverse events; USFDA, US Food and Drug Administration.
