# Supplementary material for: Efficacy of Probiotics for Irritable Bowel Syndrome: A Systematic Review and Network Meta-Analysis
Source: Front Cell Infect Microbiol. 2022 Apr 1;12:859967. doi: 10.3389/fcimb.2022.859967 (PMC9010660; doi:10.3389/fcimb.2022.859967)
Supplement: Supplementary file 1 [file DataSheet_1.pdf]

Figure S1

(a)

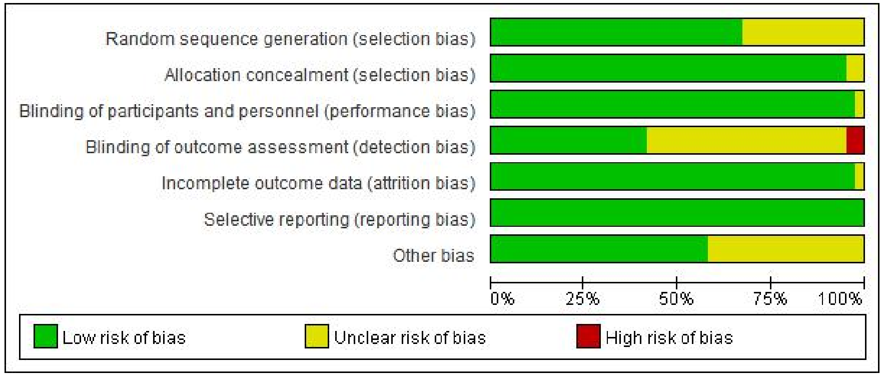

(b)

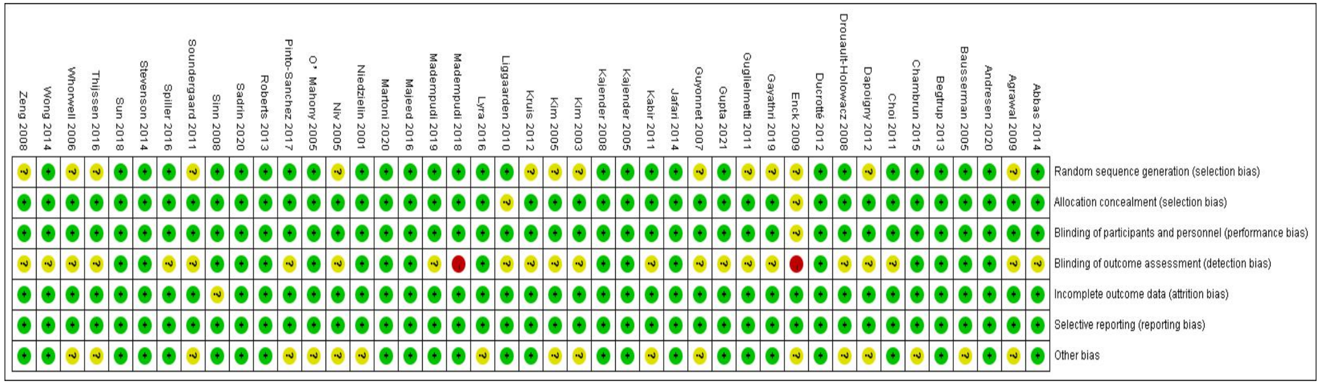

(a) Risk of bias graph: judgements about each risk of bias item presented as percentages across all included studies.

(b) Risk of bias summary: review authors' judgements about each risk of bias item for each included study.

Figure S2: Forest plot and the global inconsistency test result (symptom relief rate).
(chi^2=1.14, p=0.285)

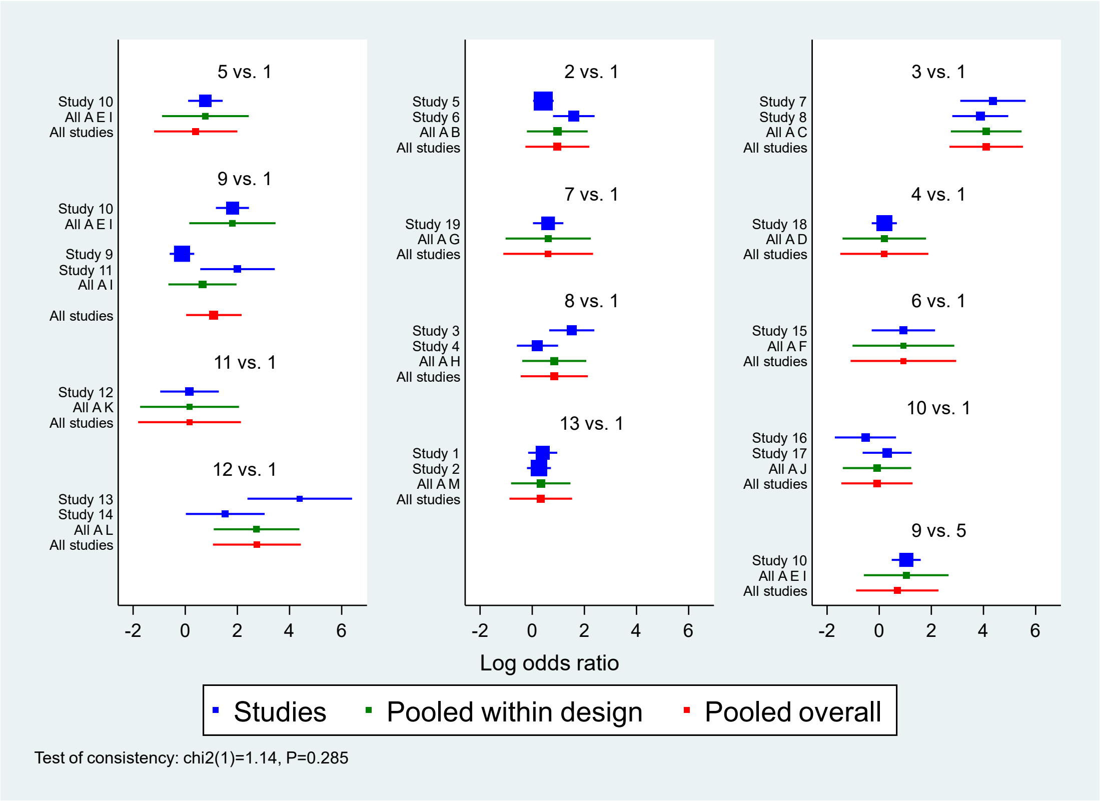

Label: PLA=1=A B.bifidum=2=B B.coagulans=3=C B.infantis=4=D B.lactis=5=E B.longum=6=F C.butyricum=7=G E.coli=8=H L.acidophilus=9=I L.casei=10=J L.GG =11=K L.plantarum=12=L S.cerevisiae=13=M

Table S1: Local inconsistency test (symptom relief rate)

Side	Direct Coef.	Direct Std. Err.	Indirect Coef.	Indirect Std. Err.	Difference Coef.	Difference Std. Err.	P>|z|	tau
A B	.	.	.	.	.	.	.	.
A C	.	.	.	.	.	.	.	.
A D	.	.	.	.	.	.	.	.
A E *	.7726241	.849371	-1.526387	1.963843	2.299011	2.151114	0.285	.7793094
A F	.	.	.	.	.	.	.	.
A G	.	.	.	.	.	.	.	.
A H	.	.	.	.	.	.	.	.
A I	.	.	.	.	.	.	.	.
A J	.	.	.	.	.	.	.	.
A K	.	.	.	.	.	.	.	.
A L	.	.	.	.	.	.	.	.
A M	.	.	.	.	.	.	.	.
E I *	1.036593	.8291856	-1.262418	1.989554	2.299011	2.151114	0.285	.7793094

Label: PLA=1=A B.bifidum=2=B B.coagulans=3=C B.infantis=4=D B.lactis=5=E B.longum=6=F C.butyricum=7=G E.coli=8=H L.acidophilus=9=I L.casei=10=J L.GG =11=K L.plantarum=12=L S.cerevisiae=13=M

Table S2: SUCRA analysis: MeanRank table (symptom relief rate)

Treatm~t	SUCRA	PrBest	MeanRank
A	21.9	0.0	10.4
B	58.3	0.0	6.0
C	99.0	88.6	1.1
D	33.5	0.0	9.0
E	38.9	0.0	8.3
F	54.4	0.4	6.5
G	45.2	0.0	7.6
H	54.1	0.0	6.5
I	63.2	0.0	5.4
J	22.9	0.0	10.2
K	32.8	0.0	9.1
L	89.6	10.9	2.2
M	36.2	0.0	8.7

Label: PLA=1=A B.bifidum=2=B B.coagulans=3=C B.infantis=4=D B.lactis=5=E B.longum=6=F C.butyricum=7=G E.coli=8=H L.acidophilus=9=I L.casei=10=J L.GG =11=K L.plantarum=12=L S.cerevisiae=13=M

Figure S3: SUCRA analysis: MeanRank figure (symptom relief)

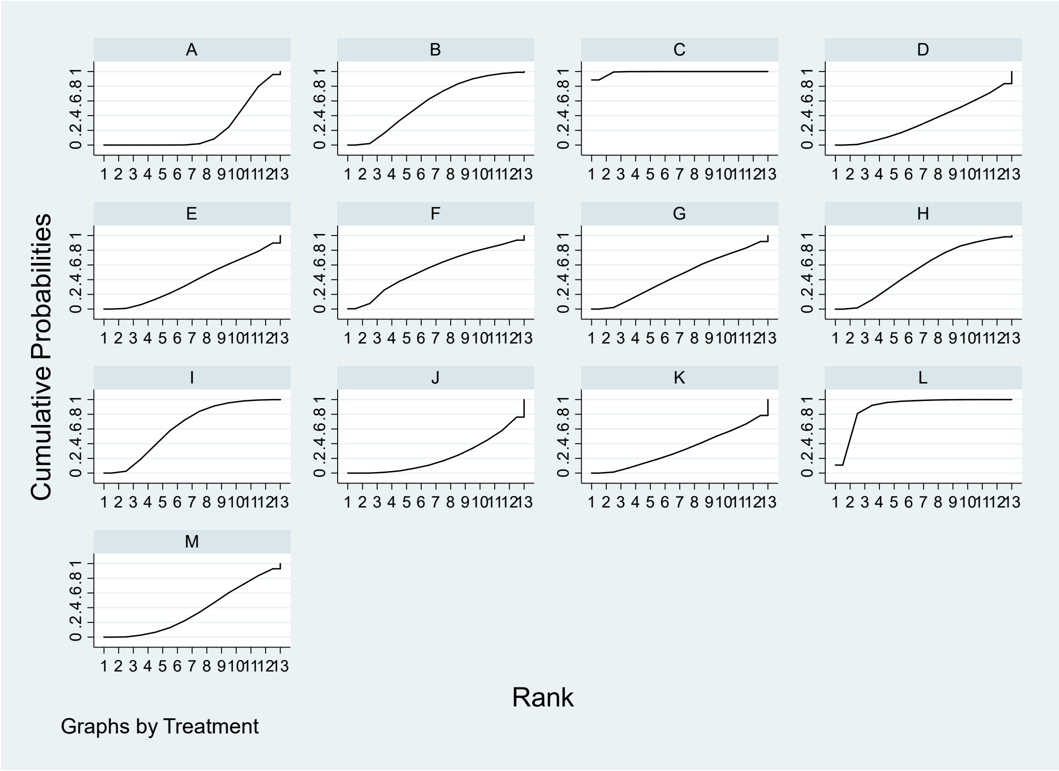

Label: PLA=1=A B.bifidum=2=B B.coagulans=3=C B.infantis=4=D B.lactis=5=E B.longum=6=F C.butyricum=7=G E.coli=8=H L.acidophilus=9=I L.casei=10=J L.GG =11=K L.plantarum=12=L S.cerevisiae=13=M

Figure S4: Forest plot and the global inconsistency test result (global symptom scores).
(chi^2=3.90, p=0.048)

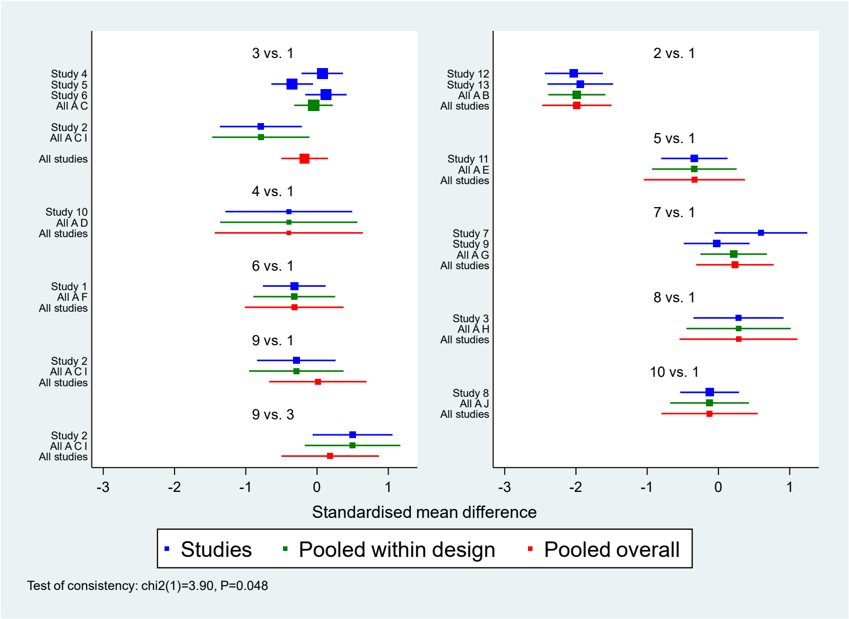

Label: PLA=1=A B.coagulans=2=B B.infantis=3=C B.longum =4=D C.butyricum=5=E L.acidophilus=6=F L.plantarum=7=G L.reuteri=8=H L.salivarius=9=I S.boulardii=10=J

Table S3: Local inconsistency test (global symptom score)

Side	Direct		Indirect		Difference			tau
	Coef.	Std. Err.	Coef.	Std. Err.	Coef.	Std. Err.	P>|z|	
A B	.	.	.	.	.	.	.	.
A C	.	.	.	.	.	.	.	.
A D	.	.	.	.	.	.	.	.
A E	.	.	.	.	.	.	.	.
A F	.	.	.	.	.	.	.	.
A G	.	.	.	.	.	.	.	.
A H	.	.	.	.	.	.	.	.
A I *	-.2907251	.3380839	1.188604	.6626518	-1.479329	.7495419	**0.048**	.1877169
A J	.	.	.	.	.	.	.	.
C I *	.4983858	.3422479	-.9809432	.6562077	1.479329	.7495419	**0.048**	.1877169

Label: PLA=1=A B.coagulans=2=B B.infantis=3=C B.longum =4=D C.butyricum=5=E L.acidophilus=6=F L.plantarum=7=G L.reuteri=8=H L.salivarius=9=I S.boulardii=10=J

Table S4: SUCRA analysis: MeanRank table (global symptom score)

Treatm~t	SUCRA	PrBest	MeanRank
A	32.5	0.0	7.1
B	100.0	99.9	1.0
C	39.3	0.0	6.5
D	63.1	0.1	4.3
E	63.5	0.0	4.3
F	62.5	0.0	4.4
G	16.7	0.0	8.5
H	17.4	0.0	8.4
I	59.2	0.0	4.7
J	45.8	0.0	5.9

Label: PLA=1=A B.coagulans=2=B B.infantis=3=C B.longum =4=D C.butyricum=5=E L.acidophilus=6=F L.plantarum=7=G L.reuteri=8=H L.salivarius=9=I S.boulardii=10=J

Figure S5: SUCRA analysis: MeanRank figure (global symptom score)

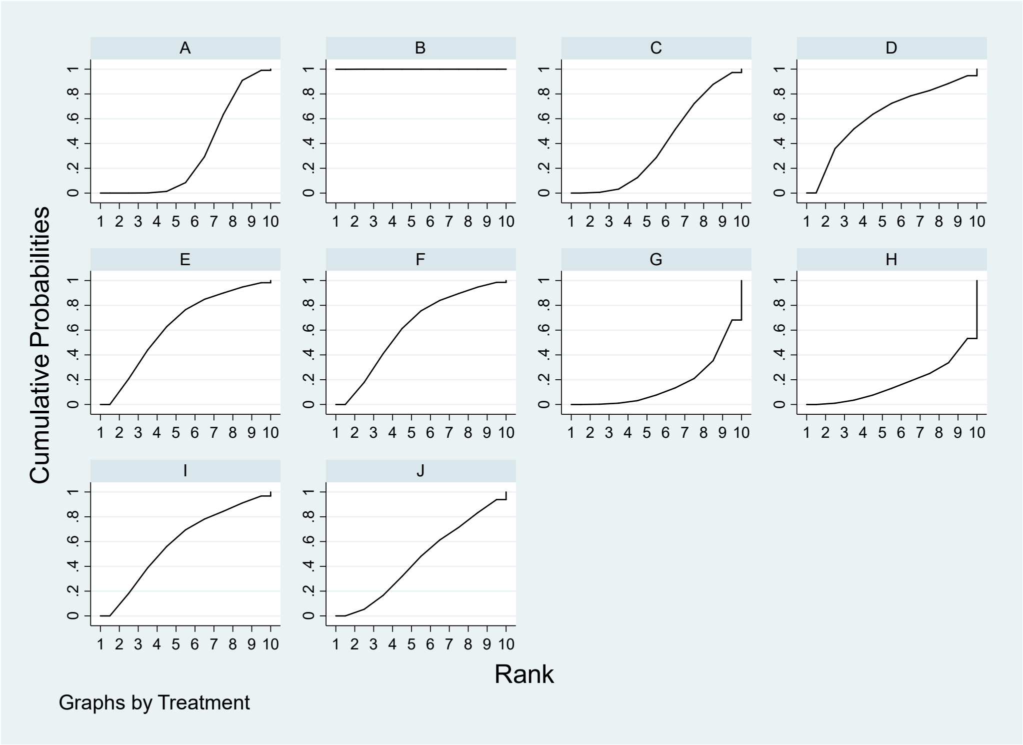

Label: PLA=1=A B.coagulans=2=B B.infantis=3=C B.longum =4=D C.butyricum=5=E L.acidophilus=6=F L.plantarum=7=G L.reuteri=8=H L.salivarius=9=I S.boulardii=10=J

Figure S6: Forest plot and the global inconsistency test result (Abdominal pain).
(chi^2=1.28, p=0.258)

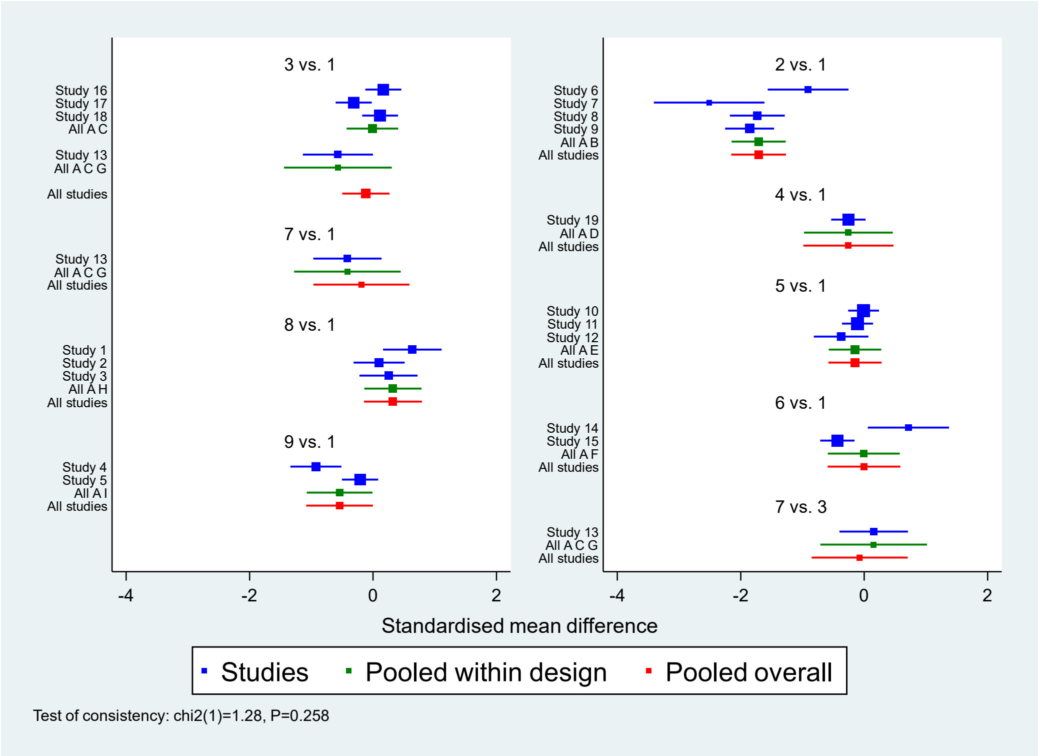

Label: PLA=1=A B.coagulans=2=B B.infantis=3=C C.butyricum=4=D L.acidophilus=5=E L.plantarum=6=F L.salivarius=7=G S.boulardii=8=H S.cerevisiae=9=I

Table S5: Local inconsistency test (Abdominal pain)

		Side	Direct			Indirect		Difference	tau
	Coef.	Std. Err.	Coef.	Std. Err.	Coef.	Std. Err.	P>|z|		
A B	.	.	.	.	.	.	.	.	
A C	.	.	.	.	.	.	.	.	
A D	.	.	.	.	.	.	.	.	
A E	.	.	.	.	.	.	.	.	
A F	.	.	.	.	.	.	.	.	
A G *	-.4148299	.4404527	.702524	.8795925	-1.117354	.9874351	0.258	.338101	
A H	.	.	.	.	.	.	.	.	
A I	.	.	.	.	.	.	.	.	
C G *	.1541163	.4411457	-.9632376	.87855	1.117354	.987435	0.258	.338101	

Label: PLA=1=A B.coagulans=2=B B.infantis=3=C C.butyricum=4=D L.acidophilus=5=E L.plantarum=6=F L.salivarius=7=G S.boulardii=8=H S.cerevisiae=9=I

Table S6: SUCRA analysis: MeanRank table (Abdominal pain)

Treatm~t	SUCRA	PrBest	MeanRank
A	31.7	0.0	6.5
B	100.0	99.8	1.0
C	45.6	0.0	5.4
D	55.4	0.1	4.6
E	48.2	0.0	5.1
F	34.7	0.0	6.2
G	49.7	0.1	5.0
H	8.2	0.0	8.3
I	76.6	0.0	2.9

Label: PLA=1=A B.coagulans=2=B B.infantis=3=C C.butyricum=4=D L.acidophilus=5=E L.plantarum=6=F L.salivarius=7=G S.boulardii=8=H S.cerevisiae=9=I

Figure S7: SUCRA analysis: MeanRank figure (Abdominal pain)

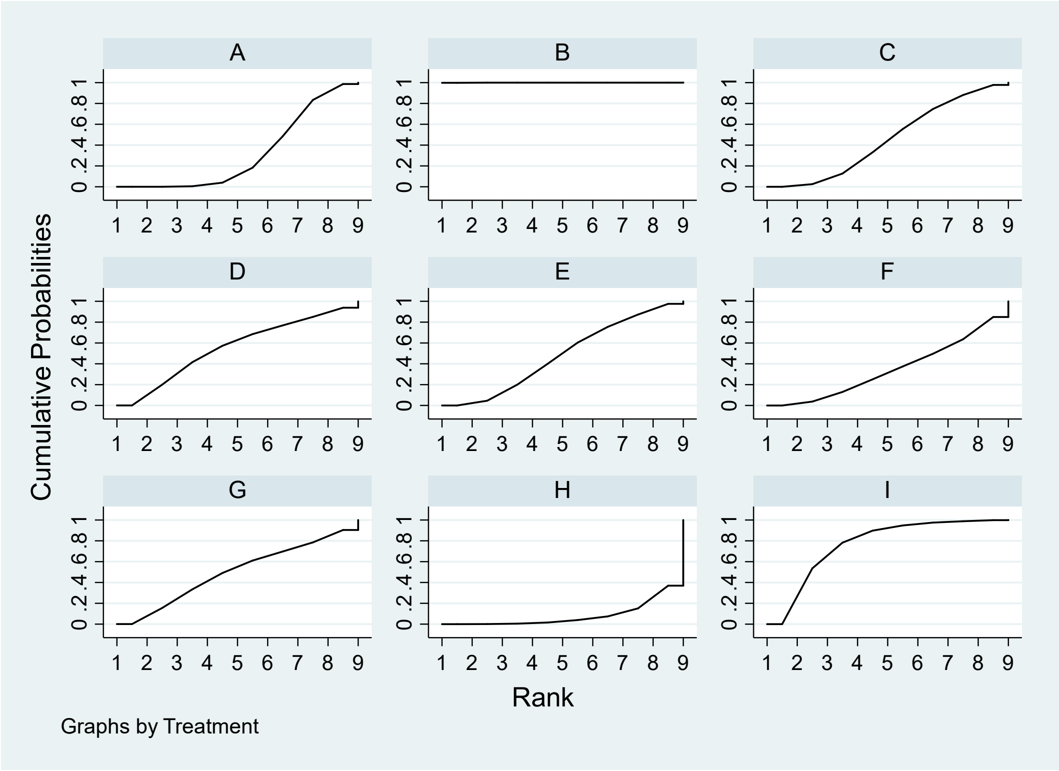

Label: PLA=1=A B.coagulans=2=B B.infantis=3=C C.butyricum=4=D L.acidophilus=5=E L.plantarum=6=F L.salivarius=7=G S.boulardii=8=H S.cerevisiae=9=I

Figure S8: Forest plot and the global inconsistency test result (Abdominal pain+treatment length).
(chi^2=0.51, p=0.475)

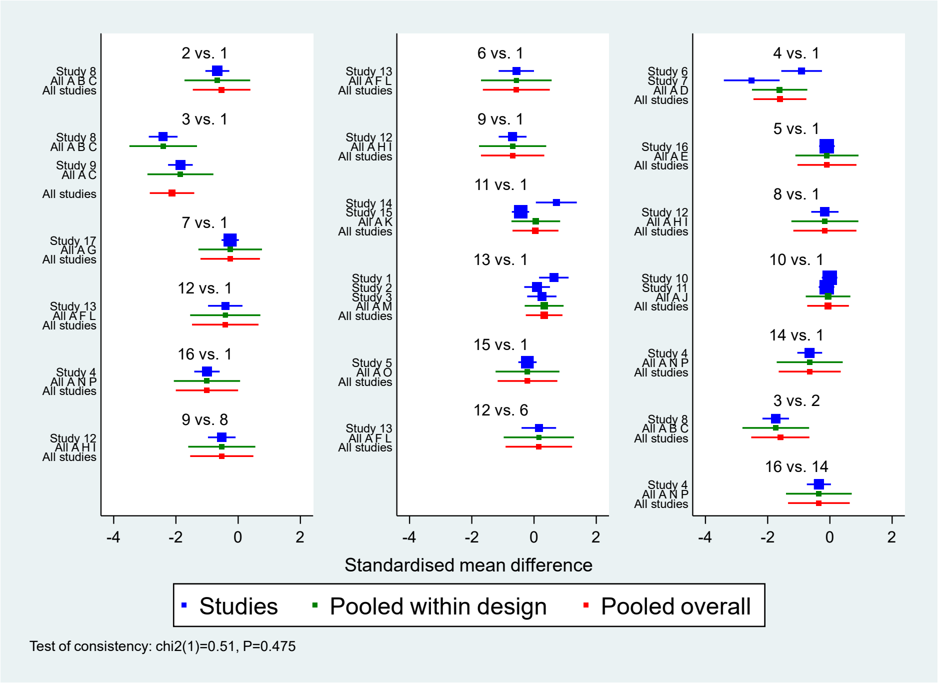

Label: PLA=1=A B.coagulans(4w)=2=B B.coagulans(8w)=3=C B.coagulans(11w/13W)=4=D B.infantis(4w)=5=E B.infantis(8w)=6=F C.butyricum(4w)=7=G L.acidophilus(4w)=8=H L.acidophilus(8w)=9=I L.acidophilus(12w)=10=J L.plantarum(3w/4W)=11=K L.salivarius(8w)=12=L S.boulardii(4w/6w)=13=M S.cerevisiae(4w)=14=N S.cerevisiae(8w)=15=O S.cerevisiae(10w)=16=P

Table S7: Local inconsistency test (Abdominal pain+treatment length).

Side	Direct Coef.	Direct Std. Err.	Indirect Coef.	Indirect Std. Err.	Difference Coef.	Difference Std. Err.	P>|z|	tau
A B *	-.6693619	.5385393	.4372391	1.446432	-1.106601	1.54874	0.475	.5015388
A C	.	.	.	.	.	.	.	.
A D	.	.	.	.	.	.	.	.
A E	.	.	.	.	.	.	.	.
A F	.	.	.	.	.	.	.	.
A G	.	.	.	.	.	.	.	.
A H	.	.	.	.	.	.	.	.
A I	.	.	.	.	.	.	.	.
A J	.	.	.	.	.	.	.	.
A K	.	.	.	.	.	.	.	.
A L	.	.	.	.	.	.	.	.
A M	.	.	.	.	.	.	.	.
A N	.	.	.	.	.	.	.	.
A O	.	.	.	.	.	.	.	.
A P	.	.	.	.	.	.	.	.
B C *	-1.740341	.5467806	-.6337399	1.437127	-1.106601	1.54874	0.475	.5015388
F L	.	.	.	.	.	.	.	.
H I	.	.	.	.	.	.	.	.
N P	.	.	.	.	.	.	.	.

Label: PLA=1=A B.coagulans(4w)=2=B B.coagulans(8w)=3=C B.coagulans(11w/13W)=4=D B.infantis(4w)=5=E B.infantis(8w)=6=F C.butyricum(4w)=7=G L.acidophilus(4w)=8=H L.acidophilus(8w)=9=I L.acidophilus(12w)=10=J L.plantarum(3w/4W)=11=K L.salivarius(8w)=12=L S.boulardii(4w/6w)=13=M S.cerevisiae(4w)=14=N S.cerevisiae(8w)=15=O S.cerevisiae(10w)=16=P

Table S8: SUCRA analysis: MeanRank table (Abdominal pain+treatment length).

Treatm~t	SUCRA	PrBest	MeanRank
A	26.6	0.0	12.0
B	56.0	0.0	7.6
C	98.2	77.7	1.3
D	90.5	17.8	2.4
E	33.6	0.0	11.0
F	57.8	0.5	7.3
G	42.7	0.1	9.6
H	37.2	0.0	10.4
I	62.5	0.7	6.6
J	31.7	0.0	11.2
K	25.9	0.0	12.1
L	50.0	0.3	8.5
M	11.6	0.0	14.3
N	60.5	0.3	6.9
O	39.8	0.0	10.0
P	75.3	2.7	4.7

Label: PLA=1=A B.coagulans(4w)=2=B B.coagulans(8w)=3=C B.coagulans(11w/13W)=4=D B.infantis(4w)=5=E B.infantis(8w)=6=F C.butyricum(4w)=7=G L.acidophilus(4w)=8=H L.acidophilus(8w)=9=I L.acidophilus(12w)=10=J L.plantarum(3w/4W)=11=K L.salivarius(8w)=12=L S.boulardii(4w/6w)=13=M S.cerevisiae(4w)=14=N S.cerevisiae(8w)=15=O S.cerevisiae(10w)=16=P

Figure S9: SUCRA analysis: MeanRank figure (Abdominal pain+treatment length).

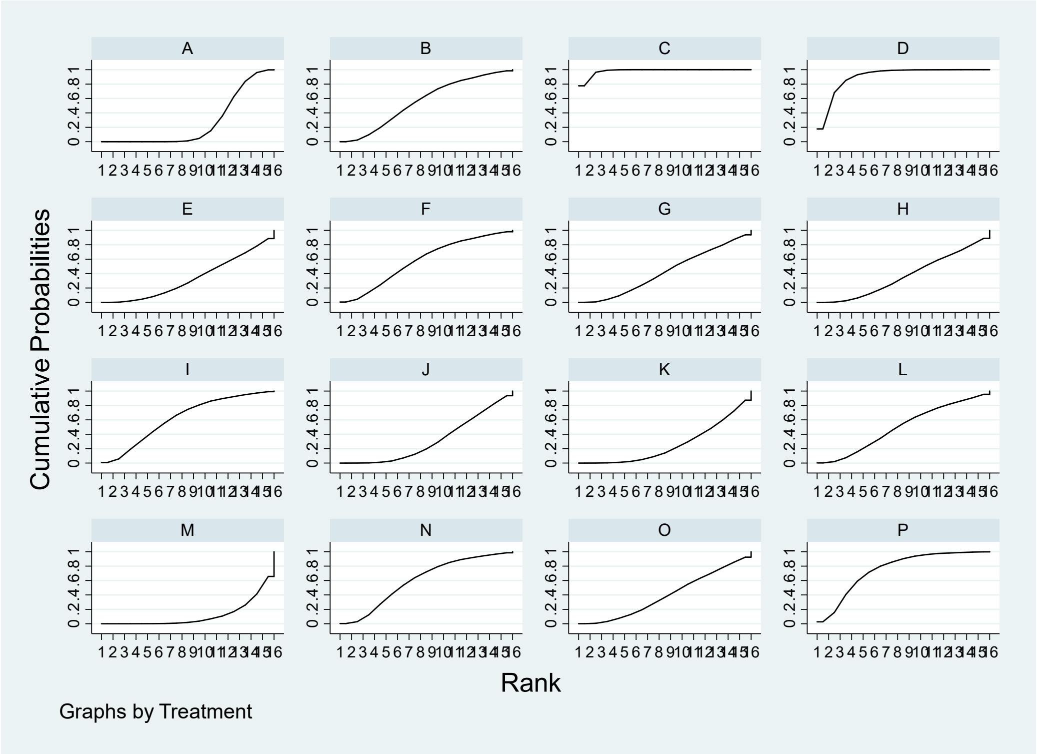

Label: PLA=1=A B.coagulans(4w)=2=B B.coagulans(8w)=3=C B.coagulans(11w/13W)=4=D B.infantis(4w)=5=E B.infantis(8w)=6=F C.butyricum(4w)=7=G L.acidophilus(4w)=8=H L.acidophilus(8w)=9=I L.acidophilus(12w)=10=J L.plantarum(3w/4W)=11=K L.salivarius(8w)=12=L S.boulardii(4w/6w)=13=M S.cerevisiae(4w)=14=N S.cerevisiae(8w)=15=O S.cerevisiae(10w)=16=P

Table S9: League table **(Abdominal pain+treatment length).**

**B.coagulans(8w)**							
-0.52 (-1.63,0.59)	**B.coagulans(11w/13w)**						
-1.12 (-2.35,0.11)	-0.60 (-1.92,0.71)	**S.cerevisiae(10w)**					
**-1.44 (-2.68,-0.19)**	-0.92 (-2.24,0.41)	-0.31 (-1.74,1.11)	**L.acidophilus(8w)**				
**-1.48 (-2.70,-0.25)**	-0.96 (-2.27,0.35)	-0.35 (-1.35,0.64)	-0.04 (-1.46,1.39)	**S.cerevisiae(4w)**			
**-1.56 (-2.85,-0.26)**	-1.04 (-2.41,0.33)	-0.43 (-1.90,1.04)	-0.12 (-1.60,1.36)	-0.08 (-1.55,1.39)	**B.infantis(8w)**		
**-1.59 (-2.53,-0.66)**	-1.07 (-2.33,0.18)	-0.47 (-1.83,0.89)	-0.16 (-1.53,1.22)	-0.12 (-1.48,1.24)	-0.04 (-1.46,1.38)	**B.coagulans(4w)**	
**-1.71 (-3.00,-0.43)**	-1.19 (-2.56,0.17)	-0.59 (-2.05,0.88)	-0.27 (-1.75,1.20)	-0.23 (-1.70,1.23)	-0.15 (-1.22,0.92)	-0.12 (-1.53,1.30)	**L.salivarius(8w)**
**-1.87 (-3.06,-0.68)**	**-1.35 (-2.63,-0.07)**	-0.75 (-2.13,0.64)	-0.43 (-1.83,0.96)	-0.39 (-1.77,0.99)	-0.31 (-1.75,1.13)	-0.28 (-1.61,1.05)	-0.16 (-1.59,1.27)
**-1.92 (-3.11,-0.72)**	**-1.40 (-2.68,-0.11)**	-0.79 (-2.18,0.59)	-0.48 (-1.88,0.92)	-0.44 (-1.82,0.95)	-0.36 (-1.80,1.08)	-0.32 (-1.66,1.01)	-0.20 (-1.64,1.23)
**-1.96 (-3.20,-0.72)**	**-1.45 (-2.77,-0.12)**	-0.84 (-2.27,0.58)	-0.53 (-1.54,0.49)	-0.49 (-1.91,0.93)	-0.41 (-1.89,1.07)	-0.37 (-1.74,1.00)	-0.25 (-1.73,1.22)
**-2.03 (-3.22,-0.84)**	**-1.51 (-2.78,-0.24)**	-0.91 (-2.29,0.47)	-0.59 (-1.98,0.80)	-0.55 (-1.93,0.82)	-0.47 (-1.91,0.96)	-0.44 (-1.76,0.89)	-0.32 (-1.75,1.11)
**-2.07 (-3.05,-1.09)**	**-1.55 (-2.63,-0.46)**	-0.94 (-2.15,0.26)	-0.63 (-1.85,0.59)	-0.59 (-1.79,0.61)	-0.51 (-1.78,0.76)	-0.47 (-1.62,0.67)	-0.36 (-1.62,0.91)
**-2.13 (-2.84,-1.41)**	**-1.61 (-2.46,-0.76)**	**-1.00 (-2.00,-0.00)**	-0.69 (-1.71,0.33)	-0.65 (-1.65,0.35)	-0.57 (-1.65,0.51)	-0.53 (-1.46,0.39)	-0.41 (-1.48,0.65)
**-2.17 (-3.20,-1.14)**	**-1.65 (-2.79,-0.52)**	-1.05 (-2.29,0.19)	-0.74 (-1.99,0.52)	-0.70 (-1.94,0.55)	-0.62 (-1.92,0.69)	-0.58 (-1.76,0.61)	-0.46 (-1.76,0.84)
**-2.45 (-3.38,-1.52)**	**-1.93 (-2.97,-0.90)**	**-1.33 (-2.49,-0.17)**	-1.01 (-2.19,0.16)	-0.97 (-2.13,0.18)	-0.89 (-2.12,0.33)	-0.86 (-1.95,0.24)	-0.74 (-1.96,0.48)

**C.butyricum(4w)**							
-0.05 (-1.40,1.31)	**S.cerevisiae(8w)**						
-0.09 (-1.49,1.30)	-0.05 (-1.45,1.35)	**L.acidophilus(4w)**					
-0.16 (-1.51,1.19)	-0.11 (-1.46,1.23)	-0.07 (-1.45,1.32)	**B.infantis(4w)**				
-0.20 (-1.36,0.97)	-0.15 (-1.32,1.02)	-0.10 (-1.32,1.11)	-0.04 (-1.20,1.13)	**L.acidophilus(12w)**			
-0.26 (-1.21,0.70)	-0.21 (-1.17,0.75)	-0.16 (-1.18,0.85)	-0.10 (-1.04,0.85)	-0.06 (-0.73,0.61)	**PLA**		
-0.30 (-1.51,0.90)	-0.26 (-1.47,0.95)	-0.21 (-1.46,1.05)	-0.14 (-1.34,1.06)	-0.11 (-1.10,0.89)	-0.05 (-0.79,0.69)	**L.plantarum(3w/4w)**	
-0.58 (-1.70,0.54)	-0.53 (-1.66,0.59)	-0.49 (-1.66,0.69)	-0.42 (-1.54,0.70)	-0.38 (-1.28,0.51)	-0.32 (-0.91,0.26)	-0.28 (-1.22,0.67)	**S.boulardii(4w/6w)**

Figure S10: Forest plot and the global inconsistency test result (Abdominal bloating).
(chi^2=0.65, p=0.420)

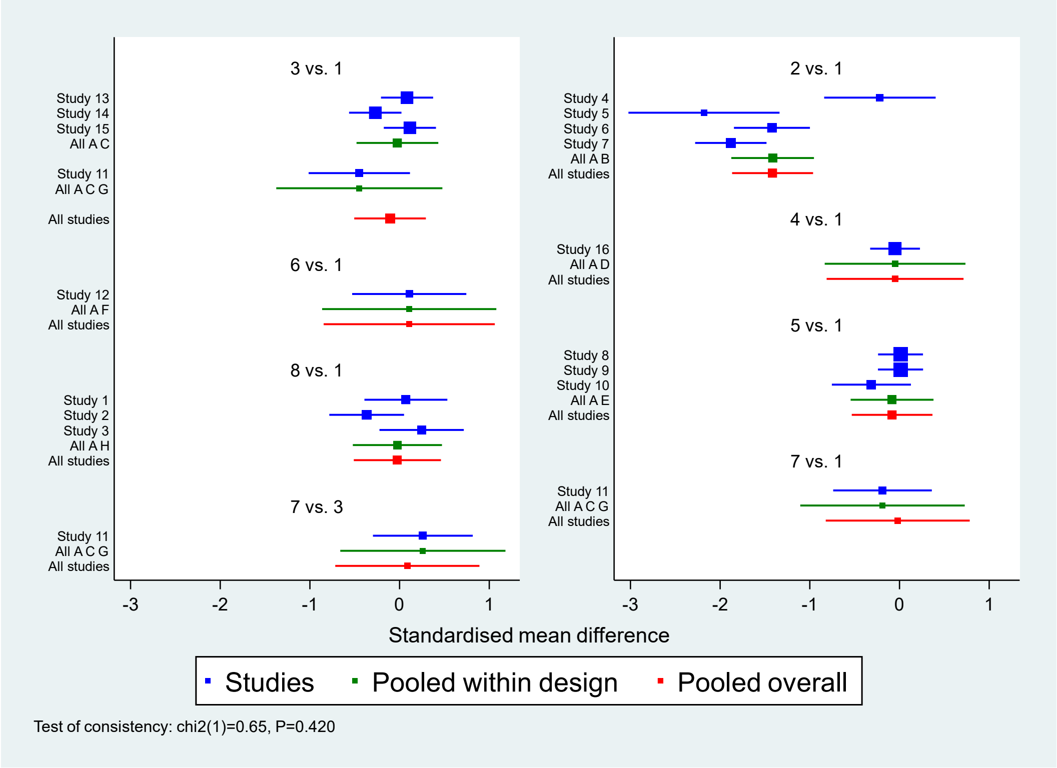

Label: PLA=1=A B.coagulans=2=B B.infantis=3=C C.butyricum=4=D L.acidophilus=5=E L.plantarum=6=F L.salivarius=7=G S.boulardii=8=H

Table S10: Local inconsistency test (Abdominal bloating).

Side	Direct		Indirect		Difference			tau
	Coef.	Std. Err.	Coef.	Std. Err.	Coef.	Std. Err.	P>|z|	
A B	.	.	.	.	.	.	.	.
A C	.	.	.	.	.	.	.	.
A D	.	.	.	.	.	.	.	.
A E	.	.	.	.	.	.	.	.
A F	.	.	.	.	.	.	.	.
A G *	-.1894072	.4679005	.6602016	.9411275	-.8496088	1.053394	0.420	.3744517
A H	.	.	.	.	.	.	.	.
C G *	.2596367	.469917	-.5899721	.9381086	.8496088	1.053394	0.420	.3744517

Label: PLA=1=A B.coagulans=2=B B.infantis=3=C C.butyricum=4=D L.acidophilus=5=E L.plantarum=6=F L.salivarius=7=G S.boulardii=8=H

Table S11: SUCRA analysis: MeanRank table (Abdominal bloating).

Treatm~t	SUCRA	PrBest	MeanRank
A	38.1	0.0	5.3
B	99.9	99.6	1.0
C	51.7	0.0	4.4
D	44.2	0.1	4.9
E	48.1	0.0	4.6
F	33.7	0.2	5.6
G	41.6	0.1	5.1
H	42.7	0.0	5.0

Label: PLA=1=A B.coagulans=2=B B.infantis=3=C C.butyricum=4=D L.acidophilus=5=E L.plantarum=6=F L.salivarius=7=G S.boulardii=8=H

Figure S11: SUCRA analysis: MeanRank figure (Abdominal bloating).

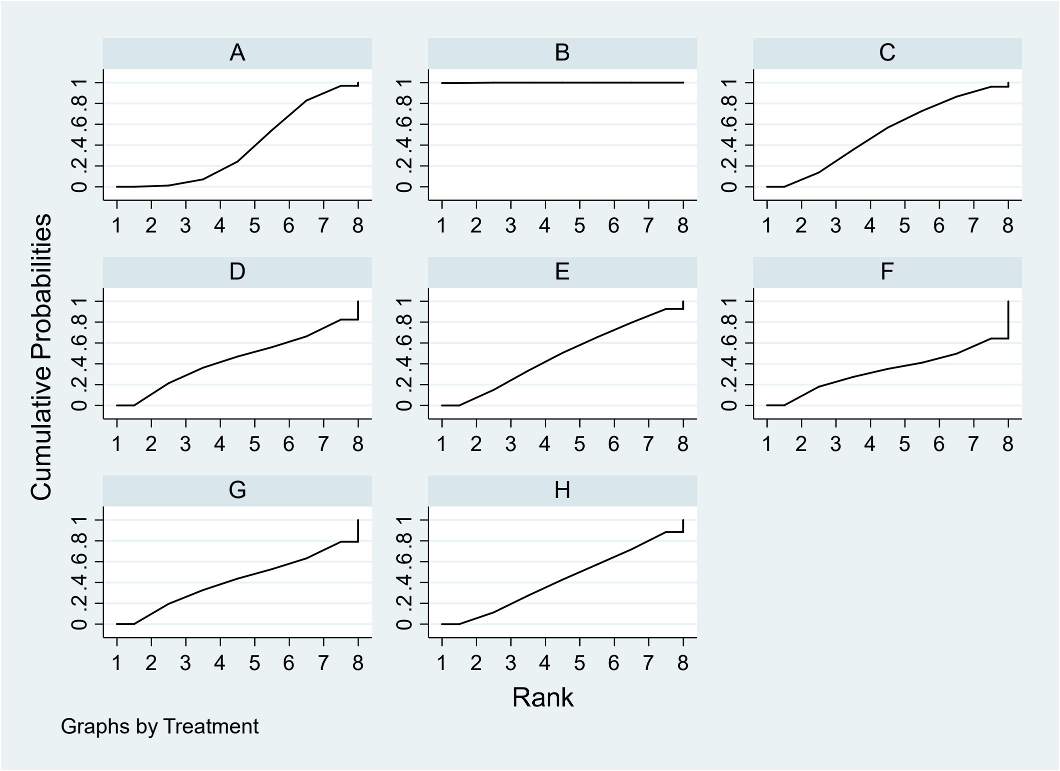

Label: PLA=1=A B.coagulans=2=B B.infantis=3=C C.butyricum=4=D L.acidophilus=5=E L.plantarum=6=F L.salivarius=7=G S.boulardii=8=H

Table S12: League table **(Abdominal bloating).**

**B.coagulans**							
**-1.31 (-1.91,-0.71)**	**B.infantis**						
**-1.33 (-1.97,-0.69)**	-0.02 (-0.62,0.58)	**L.acidophilus**					
**-1.37 (-2.25,-0.48)**	-0.06 (-0.92,0.80)	-0.03 (-0.92,0.85)	**C.butyricum**				
**-1.39 (-2.05,-0.73)**	-0.08 (-0.71,0.55)	-0.06 (-0.72,0.60)	-0.02 (-0.93,0.88)	**S.boulardii**			
**-1.40 (-2.32,-0.47)**	-0.09 (-0.89,0.72)	-0.06 (-0.98,0.86)	-0.03 (-1.14,1.08)	-0.01 (-0.94,0.93)	**L.salivarius**		
**-1.42 (-1.87,-0.96)**	-0.11 (-0.51,0.29)	-0.08 (-0.53,0.37)	-0.05 (-0.81,0.71)	-0.02 (-0.51,0.46)	-0.02 (-0.82,0.78)	**PLA**	
**-1.52 (-2.58,-0.47)**	-0.21 (-1.25,0.82)	-0.19 (-1.25,0.87)	-0.16 (-1.38,1.07)	-0.13 (-1.20,0.94)	-0.13 (-1.37,1.12)	-0.11 (-1.06,0.85)	**L.plantarum**

Table S13: SUCRA analysis: MeanRank table (Straining).

Treatm~t	SUCRA	PrBest	MeanRank
A	17.7	0.0	4.3
B	99.9	99.5	1.0
C	49.5	0.0	3.0
D	43.9	0.5	3.2
E	39.0	0.0	3.4

Label: PLA=1=A B.coagulans=2=B B.infantis=3=C L.plantarum=4=D S.boulardii=5=E

Figure S12: SUCRA analysis: MeanRank figure (Straining).

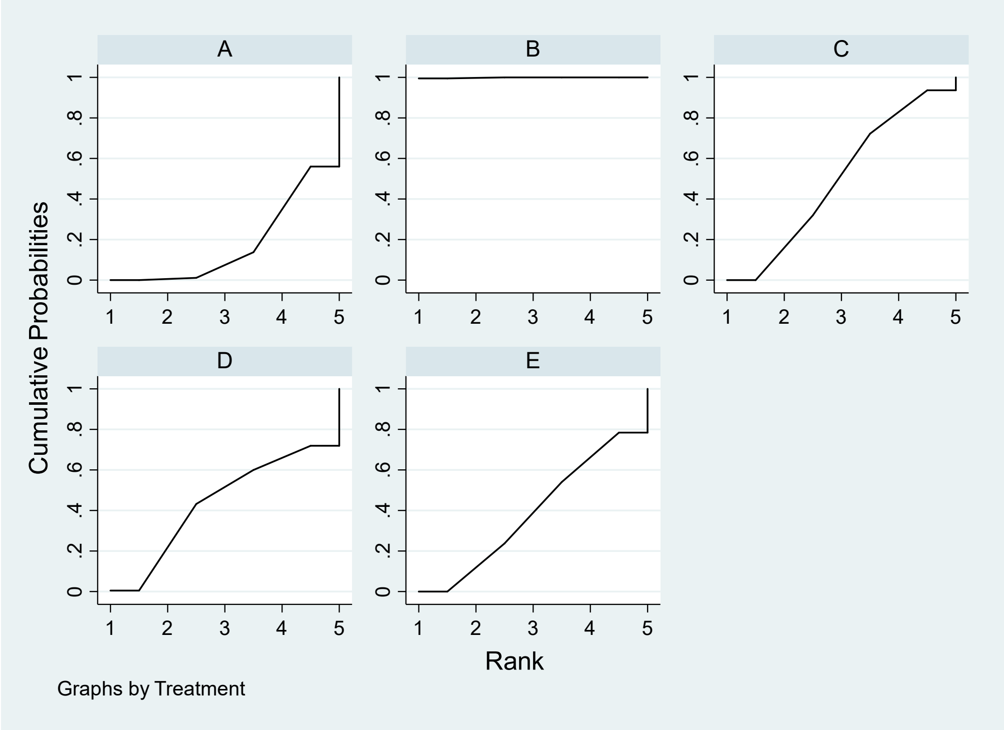

Label: PLA=1=A B.coagulans=2=B B.infantis=3=C L.plantarum=4=D S.boulardii=5=E

Table S14: League table **(Straining).**

**B.coagulans**				
**-1.11 (-1.55,-0.66)**	**B.infantis**			
**-1.11 (-1.93,-0.29)**	-0.00 (-0.79,0.79)	**L.plantarum**		
**-1.18 (-1.71,-0.64)**	-0.07 (-0.56,0.43)	-0.07 (-0.92,0.78)	**S.boulardii**	
**-1.29 (-1.63,-0.94)**	-0.18 (-0.46,0.10)	-0.18 (-0.92,0.56)	-0.11 (-0.52,0.30)	**PLA**

Figure S13: Forest plot and the global inconsistency test result (Strain scores: probiotics+length**)**
(chi^2=6.52, p= 0.0107)

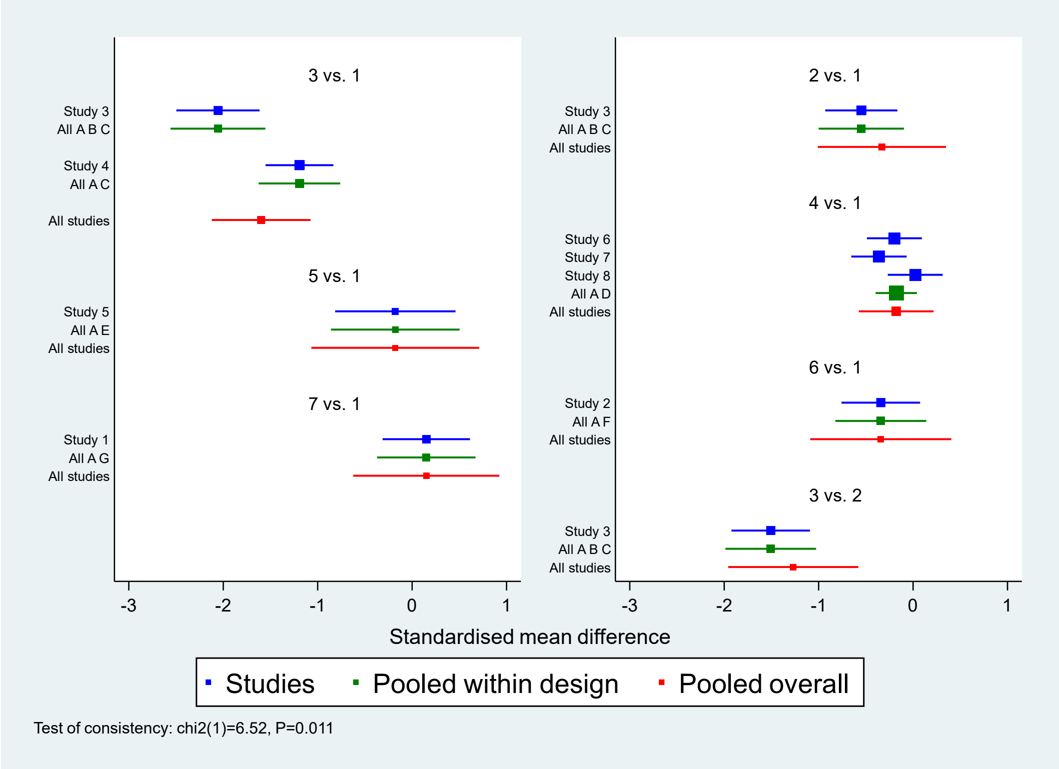

Label: *PLA=1=A* *B.coagulans(4w)=2=B* *B.coagulans(8w)=3=C* *B.infantis(4w)=4=D* *L.plantarum(3w)=5=E* *S.boulardii(4w)=6=F* *S.boulardii(6w)=7=G*

Table S15: Local inconsistency test (Strain scores: probiotics+length**)**

Side	Direct		Indirect		Difference			tau
	Coef.	Std. Err.	Coef.	Std. Err.	Coef.	Std. Err.	P>|z|	
A B *	-.5482431	.2303674	1.177715	.6267571	-1.725958	.6760362	0.011	.1227094
A C	.	.	.	.	.	.	.	.
A D	.	.	.	.	.	.	.	.
A E	.	.	.	.	.	.	.	.
A F	.	.	.	.	.	.	.	.
A G	.	.	.	.	.	.	.	.
B C *	-1.507669	.2450861	.218289	.6097797	-1.725958	.676037	0.011	.1227094

Label: *PLA=1=A B.coagulans(4w)=2=B B.coagulans(8w)=3=C B.infantis(4w)=4=D L.plantarum(3w)=5=E S.boulardii(4w)=6=F S.boulardii(6w)=7=G*

Table S16: Result of SUCRA analysis: MeanRank table **(Strain scores:** probiotics+length**)**

+--------------------------------------+

Treatm~t	SUCRA	PrBest	MeanRank
A	25.8	0.0	5.4
B	56.2	0.0	3.6
C	99.9	99.3	1.0
D	46.9	0.0	4.2
E	43.6	0.2	4.4
F	56.9	0.4	3.6
G	20.7	0.0	5.8

Label: *PLA=1=A B.coagulans(4w)=2=B B.coagulans(8w)=3=C B.infantis(4w)=4=D L.plantarum(3w)=5=E S.boulardii(4w)=6=F S.boulardii(6w)=7=G*

Figure S14: Result of SUCRA analysis: MeanRank figure **(Strain scores:** probiotics+length**)**

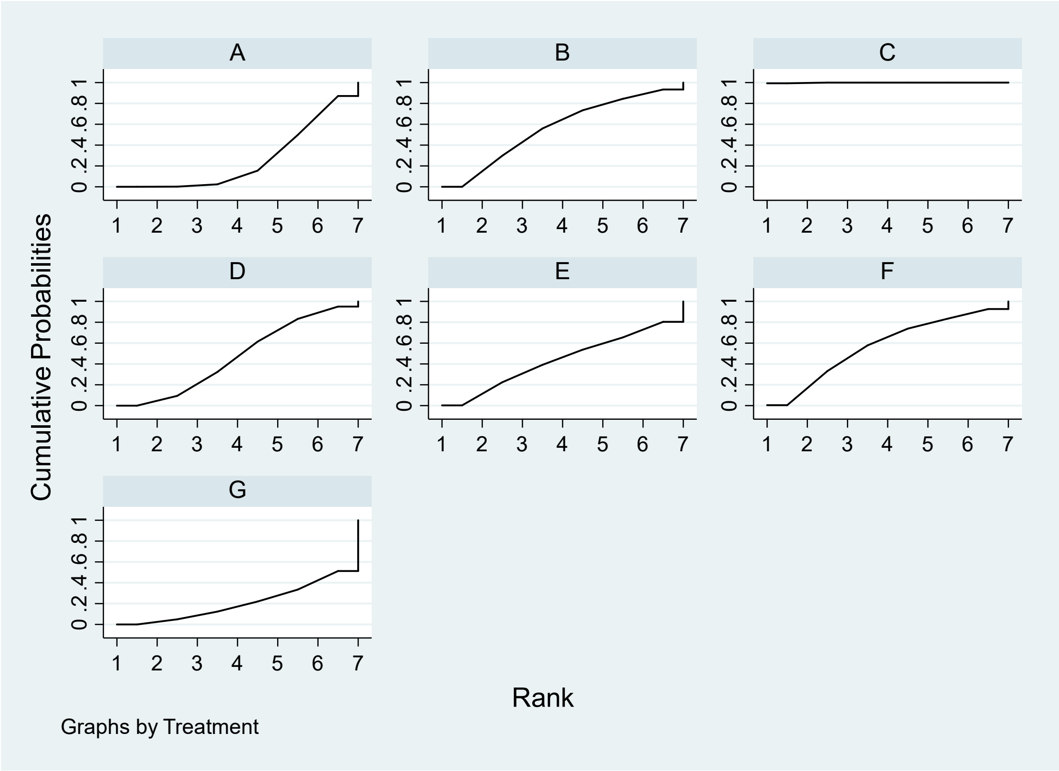

Label: *PLA=1=A B.coagulans(4w)=2=B B.coagulans(8w)=3=C B.infantis(4w)=4=D L.plantarum(3w)=5=E S.boulardii(4w)=6=F S.boulardii(6w)=7=G*

Table S17: league table (Strain scores: probiotics+length**)**

B.coagulans(8w)						
**-1.26 (-2.17,-0.34)**	*S.boulardii(4w)*					
**-1.27 (-1.96,-0.58)**	-0.01 (-1.02,1.00)	*B.coagulans(4w)*				
**-1.42 (-2.08,-0.76)**	-0.16 (-1.01,0.68)	-0.15 (-0.94,0.63)	*B.infantis(4w)*			
**-1.42 (-2.45,-0.39)**	-0.16 (-1.33,1.00)	-0.15 (-1.27,0.97)	-0.00 (-0.97,0.97)	*L.plantarum(3w)*		
**-1.60 (-2.12,-1.08)**	-0.34 (-1.09,0.41)	-0.33 (-1.01,0.35)	-0.18 (-0.57,0.22)	-0.18 (-1.07,0.71)	PLA	
**-1.75 (-2.68,-0.81)**	-0.49 (-1.57,0.58)	-0.48 (-1.51,0.55)	-0.33 (-1.20,0.54)	-0.33 (-1.51,0.85)	-0.15 (-0.92,0.62)	*S.boulardii(6w)*

Label: *PLA=1=A B.coagulans(4w)=2=B B.coagulans(8w)=3=C B.infantis(4w)=4=D L.plantarum(3w)=5=E S.boulardii(4w)=6=F S.boulardii(6w)=7=G*

Table S18: Result of SUCRA analysis: MeanRank table **(QOL)**

Treatm~t	SUCRA	PrBest	MeanRank
A	30.3	0.5	3.8
B	40.8	9.7	3.4
C	44.0	6.9	3.2
D	79.8	59.8	1.8
E	55.2	23.1	2.8

Label: PLA=1=A C.butyricum=2=B L.acidophilus=3=C L.plantarum=4=D S.boulardii=5=E

Figure S15: Result of SUCRA analysis: MeanRank figure **(QOL)**

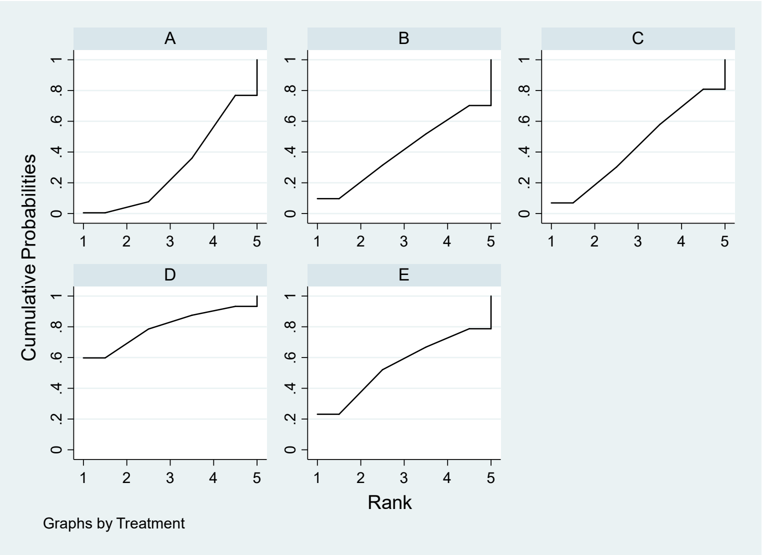

Label: PLA=1=A C.butyricum=2=B L.acidophilus=3=C L.plantarum=4=D S.boulardii=5=E

Table S19: league table (QOL)

**L.plantarum**				
0.20 (-0.54,0.93)	**S.boulardii**			
0.27 (-0.33,0.88)	0.08 (-0.49,0.64)	**L.acidophilus**		
0.28 (-0.39,0.96)	0.09 (-0.55,0.72)	0.01 (-0.46,0.49)	**C.butyricum**	
0.33 (-0.22,0.87)	0.13 (-0.37,0.63)	0.05 (-0.21,0.32)	0.04 (-0.35,0.44)	**PLA**

Table S20: Result of SUCRA analysis: MeanRank table **(Adverse events)**

Treatm~t	SUCRA	PrBest	MeanRank
A	68.8	0.0	3.5
B	33.3	0.0	6.3
C	20.6	0.9	7.4
D	19.2	1.6	7.5
E	51.2	0.7	4.9
F	98.1	85.8	1.2
G	62.2	9.2	4.0
H	50.2	1.9	5.0
I	46.4	0.0	5.3

Label: PLA=1=A B.bifidum=2=B B.longum=3=C C.butyricum=4=D E.coli=5=E L.acidophilus=6=F L.reuteri=7=G S.boulardii=8=H S.cerevisiae=9=I

Figure S16: Result of SUCRA analysis: MeanRank figure **(Adverse events)**

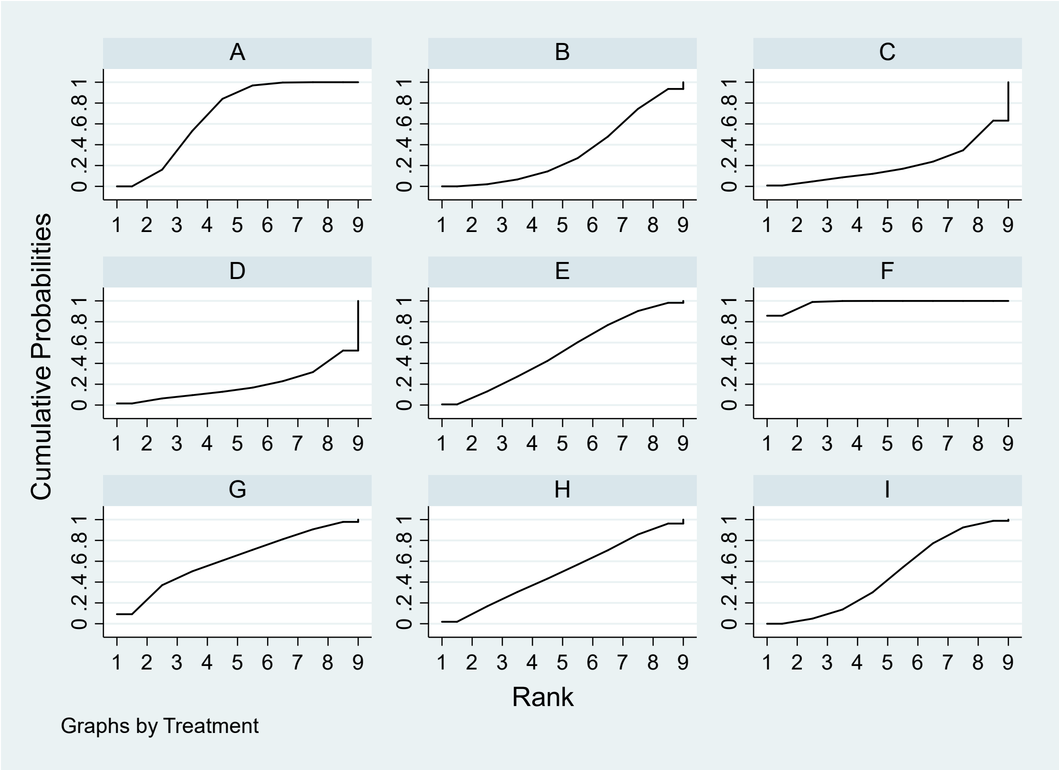

Label: PLA=1=A B.bifidum=2=B B.longum=3=C C.butyricum=4=D E.coli=5=E L.acidophilus=6=F L.reuteri=7=G S.boulardii=8=H S.cerevisiae=9=I

Table S21: league table (Adverse events)

**L.acidophilus**								
**0.47 (0.32,0.67)**	**PLA**							
0.47 (0.15,1.44)	1.00 (0.34,2.91)	**L.reuteri**						
**0.38 (0.17,0.85)**	0.82 (0.40,1.68)	0.82 (0.23,2.96)	**E.coli**					
**0.37 (0.14,0.97)**	0.79 (0.33,1.94)	0.79 (0.20,3.20)	0.97 (0.31,3.05)	**S.boulardii**				
**0.35 (0.19,0.65)**	0.76 (0.46,1.24)	0.76 (0.23,2.45)	0.92 (0.39,2.21)	0.95 (0.34,2.64)	**S.cerevisiae**			
**0.29 (0.14,0.60)**	0.62 (0.33,1.17)	0.62 (0.18,2.14)	0.76 (0.29,1.97)	0.78 (0.26,2.33)	0.82 (0.37,1.82)	**B.bifidum**		
**0.18 (0.04,0.76)**	0.39 (0.10,1.56)	0.39 (0.07,2.24)	0.48 (0.10,2.27)	0.49 (0.09,2.55)	0.51 (0.12,2.24)	0.63 (0.14,2.89)	**B.longum**	
**0.17 (0.03,0.88)**	0.35 (0.07,1.80)	0.35 (0.05,2.48)	0.43 (0.07,2.56)	0.45 (0.07,2.85)	0.47 (0.09,2.56)	0.57 (0.10,3.28)	0.91 (0.11,7.74)	**C.butyricum**

Figure S17: Result of SUCRA analysis: MeanRank figure **(global symptom scores:** *B. coagulans vs. Probiotic combinations***)**

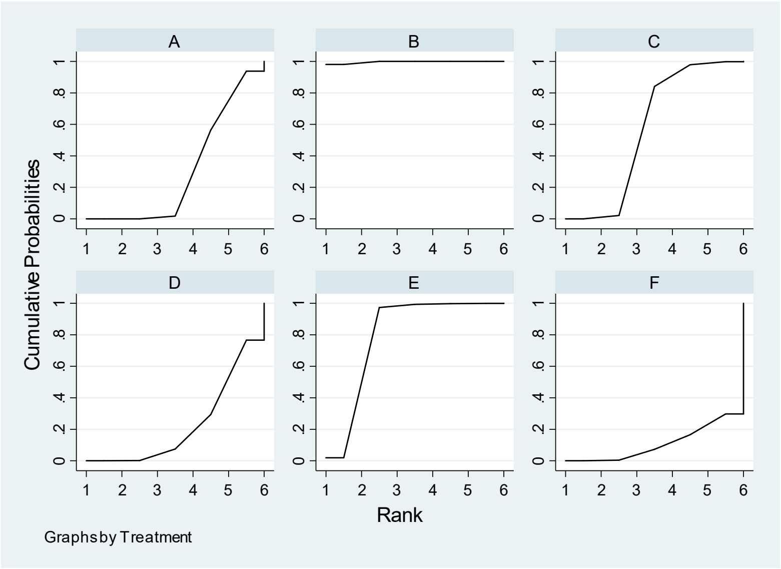

Labels: PLA=A *B. coagulans*=B 1B1L1S=C 1B2L=D 1B2L1S=E 3B4L1S=F

Table S22: league table (global symptom scores: *B. coagulans vs. Probiotic combinations*)

B					
**-0.90 (-1.74,-0.05)**	E				
**-1.75 (-2.14,-1.36)**	**-0.85 (-1.68,-0.03)**	C			
**-1.99 (-2.29,-1.68)**	**-1.09 (-1.88,-0.30)**	**-0.24 (-0.48,-0.00)**	A		
**-2.08 (-2.58,-1.58)**	**-1.19 (-2.07,-0.30)**	-0.34 (-0.80,0.13)	-0.09 (-0.49,0.30)	D	
**-2.35 (-3.20,-1.50)**	**-1.46 (-2.57,-0.34)**	-0.60 (-1.43,0.22)	-0.36 (-1.16,0.43)	-0.27 (-1.16,0.62)	F

Labels: PLA=A *B. coagulans*=B 1B1L1S=C 1B2L=D 1B2L1S=E 3B4L1S=F

Figure S18: Result of SUCRA analysis: MeanRank figure **(Symptom relief rate:** *B. coagulans vs. Probiotic combinations***)**

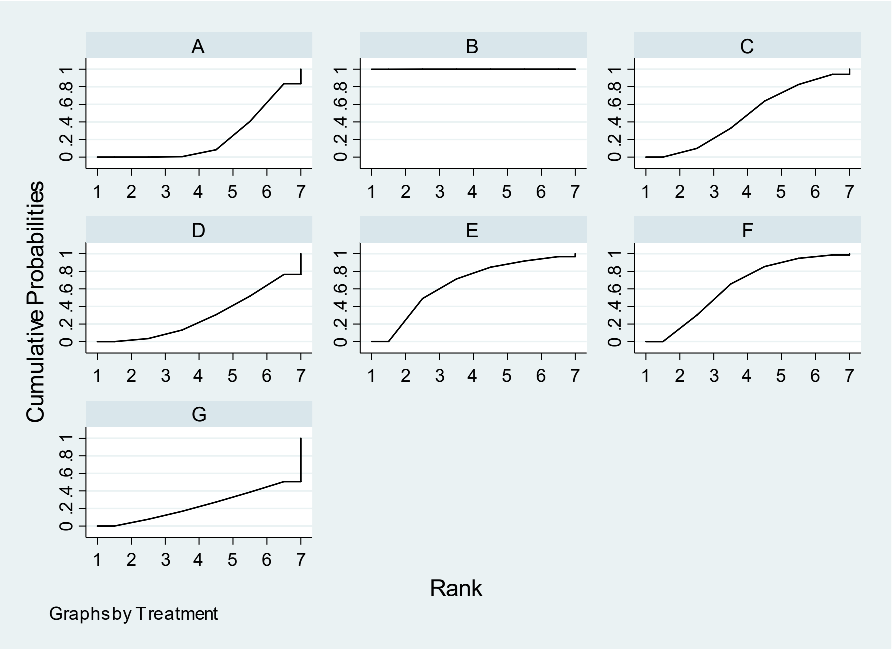

Labels: PLA=A *B. coagulans*=B 1B1L1S=C 1B2L=D 1B2L1P=E 1B2L1S=F 3B4L1S=G

Table S23: league table (Symptom relief rate: *B. coagulans vs. Probiotic combinations***)**

B						
**3.02 (1.06,4.98)**	E					
**3.21 (1.56,4.86)**	0.19 (-1.69,2.07)	F				
**3.61 (2.03,5.19)**	0.59 (-1.23,2.41)	0.40 (-1.08,1.88)	C			
**4.02 (2.35,5.69)**	1.00 (-0.90,2.90)	0.81 (-0.77,2.39)	0.41 (-1.09,1.92)	D		
**4.33 (1.90,6.75)**	1.31 (-1.28,3.89)	1.12 (-1.25,3.48)	0.72 (-1.60,3.03)	0.30 (-2.07,2.68)	G	
**4.10 (2.87,5.33)**	1.08 (-0.44,2.61)	0.89 (-0.21,1.99)	0.49 (-0.50,1.49)	0.08 (-1.05,1.21)	-0.22 (-2.31,1.87)	A

Labels: PLA=A *B. coagulans*=B 1B1L1S=C 1B2L=D 1B2L1P=E 1B2L1S=F 3B4L1S=G

Figure S19: Result of SUCRA analysis: MeanRank figure **(Abdominal pain:** *B. coagulans vs. Probiotic combinations***)**

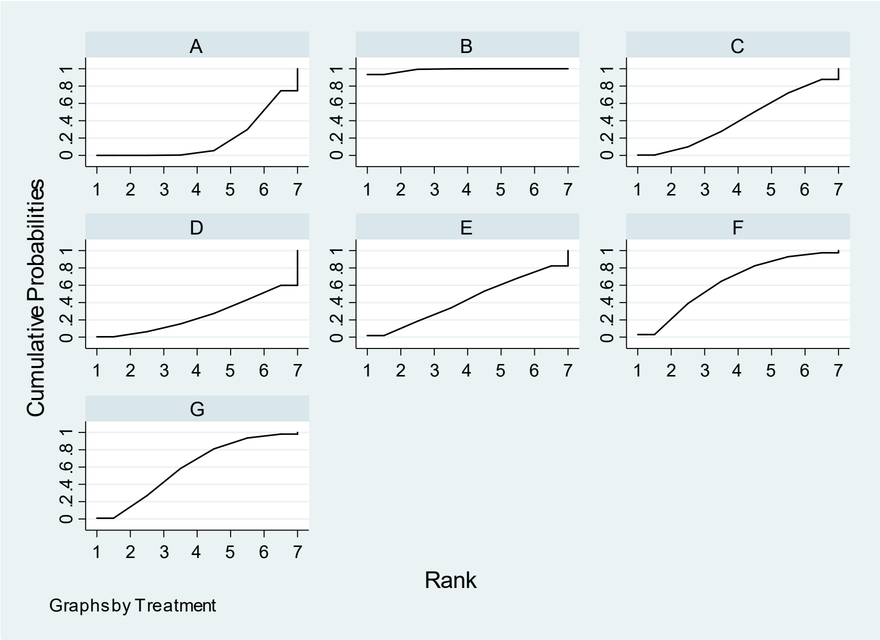

Labels: PLA=A *B. coagulans*=B 1B1L1S=C 1B2L=D 1B2L1P=E 1B2L1S=F 3B4L1S=G

Table S24: league table (Abdominal pain: *B. coagulans vs. Probiotic combinations***)**

B						
-1.01 (-2.07,0.05)	F					
**-1.08 (-2.03,-0.13)**	-0.07 (-1.20,1.07)	G				
**-1.36 (-2.66,-0.06)**	-0.35 (-1.79,1.09)	-0.28 (-1.64,1.08)	E			
**-1.40 (-2.43,-0.36)**	-0.38 (-1.59,0.82)	-0.32 (-1.43,0.79)	-0.04 (-1.46,1.39)	C		
**-1.72 (-3.01,-0.43)**	-0.71 (-2.14,0.72)	-0.64 (-1.99,0.71)	-0.36 (-1.98,1.25)	-0.33 (-1.74,1.08)	D	
**-1.72 (-2.33,-1.11)**	-0.71 (-1.58,0.16)	-0.64 (-1.37,0.08)	-0.36 (-1.51,0.79)	-0.33 (-1.16,0.51)	-0.00 (-1.13,1.13)	A

Labels: PLA=A *B. coagulans*=B 1B1L1S=C 1B2L=D 1B2L1P=E 1B2L1S=F 3B4L1S=G

Figure S20: Result of SUCRA analysis: MeanRank figure **(Abdominal bloating:** *B. coagulans vs. Probiotic combinations***)**

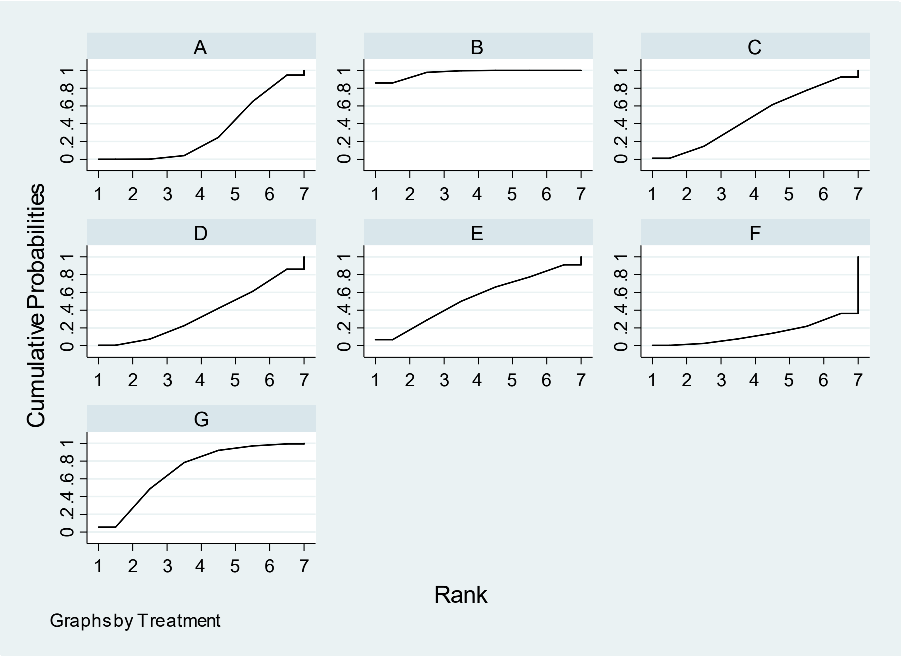

Labels: PLA=A *B. coagulans*=B 1B1L1S=C 1B2L=D 1B2L1P=E 1B2L1S=F 3B4L1S=G

Table S25: league table (Abdominal bloating: *B. coagulans vs. Probiotic combinations***)**

**B**						
-0.77 (-1.77,0.23)	G					
-1.04 (-2.42,0.34)	-0.27 (-1.71,1.18)	E				
**-1.17 (-2.27,-0.08)**	-0.41 (-1.58,0.76)	-0.14 (-1.65,1.37)	C			
**-1.37 (-2.46,-0.29)**	-0.61 (-1.77,0.56)	-0.34 (-1.84,1.17)	-0.20 (-1.44,1.04)	D		
**-1.41 (-2.05,-0.77)**	-0.64 (-1.41,0.12)	-0.38 (-1.60,0.85)	-0.24 (-1.12,0.65)	-0.04 (-0.91,0.84)	A	
**-1.97 (-3.48,-0.47)**	-1.20 (-2.77,0.36)	-0.94 (-2.77,0.90)	-0.80 (-2.42,0.83)	-0.60 (-2.22,1.02)	-0.56 (-1.92,0.80)	F

Labels: PLA=A *B. coagulans*=B 1B1L1S=C 1B2L=D 1B2L1P=E 1B2L1S=F 3B4L1S=G

Table S26: league table (Abdominal pain: Comparisons of different strains of *B. coagulans*)

B.coagulans MTCC5856			
-0.71 (-1.65,0.23)	*B.coagulans* Unique IS2		
**-1.60 (-2.71,-0.49)**	**-0.89 (-1.61,-0.17)**	*B.coagulans* LBSC	
**-2.51 (-3.41,-1.62)**	**-1.80 (-2.10,-1.51)**	**-0.91 (-1.56,-0.25)**	PLA

Table S27: league table (Abdominal bloating: Comparisons of different strains of *B. coagulans*)

B.coagulans MTCC5856		
-0.52 (-1.59,0.55)	*B.coagulans* Unique IS2	
**-2.18 (-3.15,-1.21)**	**-1.66 (-2.11,-1.21)**	PLA

Table S28: league table (Straining scores: Comparisons of different strains of *B. coagulans*)

B.coagulans Unique IS2		
-0.56 (-1.52,0.39)	*B.coagulans* LBSC	
**-1.41 (-1.88,-0.95)**	**-0.85 (-1.68,-0.02)**	PLA
